# Unified Simulation Platform for Optical Tweezers and Optofluidic Force Induction

**DOI:** 10.1021/acsphotonics.5c00254

**Published:** 2025-03-22

**Authors:** Ulrich Hohenester, Marko Šimić, Raphael Hauer, Lorenz Huber, Christian Hill

**Affiliations:** † Institute of Physics, University of Graz, Universitätsplatz 5, 8010 Graz, Austria; ‡ Brave Analytics GmbH, 8010 Graz, Austria; § Gottfried Schatz Research Center, Division of Biophysics, Medical University of Graz, Neue Stiftingtalstraße 2, 8010 Graz, Austria

**Keywords:** optical tweezers, optofluidic force induction, simulations, T-matrices

## Abstract

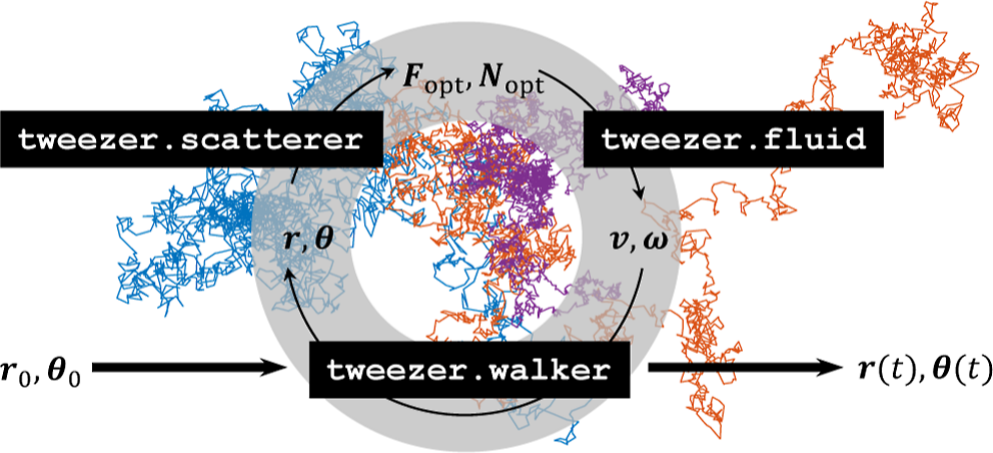

Optical tweezers utilize the forces exerted by focused laser beams to trap particles. In optofluidic force induction (OF2i), the forces exerted by a weakly focused laser beam trap particles in the transverse directions and push them in the laser propagation direction, which can be utilized for optical nanoparticle characterization with single-particle sensitivity. Here, we present a unified approach for the simulation of nanoparticles propagating in the presence of fluidic and optical forces, which can be used for both optical tweezers and OF2i simulations. We demonstrate the working principle at a number of selected examples and provide the simulation software as an add-on to our generic Maxwell solver NANOBEM that is based on a boundary element method approach.

## 1 Introduction

Optical tweezers have become an indispensable tool in various fields of research, ranging from physics over biochemistry to (nano)medicine.^1−6^ For many years, the field has been pioneered by Arthur Ashkin,^7^ who received in 2018 the Nobel Prize in Physics for his work on “optical tweezers and their application to biological systems”. The working principle is the transfer of momentum from light to matter. For tightly focused laser beams, typical optical forces are in the pico-Newton regime, which suffices to trap and manipulate particles with diameters ranging from a few tens of nanometers to a few micrometers.

Optofluidic force induction (OF2i) is an optical nanoparticle characterization scheme that exploits the optical tweezers principle, however, with a weakly focused laser beam and for trapping only in the transverse directions.^8−10^ In OF2i, particles to be analyzed are pumped through a microfluidic capillary, and the laser beam propagating in the same direction as the particle flow exerts optical forces on the particles, which lead to size-dependent velocity enhancements that can be measured and used to infer the size of single particles or the size distributions of particle ensembles. OF2i can serve as a process analytical technology^10^ and can be combined with complementary techniques, such as Raman scattering or inductively coupled plasma–mass spectrometry, to obtain detailed information about the analytes.^11^

In ref (9), we have presented a theoretical description of OF2i based on the simulation software that we had developed over the past years. It took us a while to realize that our approach had a strong overlap with that of optical tweezers. References (3 and 12) and the papers cited therein provide detailed information about the implementation of optical tweezer simulations, as well as links to the existing software such as the optical tweezer toolbox OTT^13^ or the optical tweezer software OTS.^3^ These overarching design criteria for nanoparticle simulations based on optical and fluidic forces have been our initial trigger to seek a unified simulation platform suitable for a wide range of applications.

A further impetus has come from independent software developments by one of the authors of this paper in the context of the NANOBEM toolbox, which is a generic Maxwell solver for nanoparticles based on the boundary element method (BEM).^14−16^ This toolbox and its preceding version^17^ have been extensively used over a decade for the simulation of plasmonic nanoparticles, but two recent extensions brought it closer to optical tweezer simulations. In ref (18), we presented a unified simulation platform for interference microscopies, which has been achieved by implementing generic classes for focusing of laser beams and for simulating imaging using the framework of Richards and Wolf.^19^ Second, the NANOBEM toolbox has taken part in the T-matrix initiative of Carsten Rockstuhl and co-workers,^20^ which suggests a unified storage format. Within this initiative, our toolbox has been modified such that it can now easily compute T-matrices for a variety of different nanoparticle geometries. Alternatively, one can also load T-matrices computed with different Maxwell solvers into the toolbox.

With these software developments, time is ripe for a unified platform for the simulation of particles propagating in the presence of optical and fluidic forces, which can be used for both optical tweezers and OF2i simulations. In this paper, we describe an implementation within NANOBEM that is based on a T-matrix description for the scatterers, from which one can compute optical forces, optical cross sections, and optical far fields. We provide a number of functions and classes for the computation of strongly and weakly focused laser beams, but the software is flexible enough to cope with any type of user-defined electromagnetic fields. We have also implemented classes for simulating particle trajectories in the presence of optical and fluidic forces, including Brownian motion.

This article has been organized as follows. In the Theory section, we summarize the theoretical framework underlying our software. The simulation section gives a short overview of the design criteria and functionalities of the toolbox. Selected results for optical tweezers and OF2i are presented in the Results and Discussion section, and a brief summary is given in the Summary and Outlook section. Some of the technical details are presented in the Appendix. The paper comes along with a Supporting Information, where we discuss how to install the toolbox and give a more technical and detailed description of the toolbox features as well as with the simulation software that is fully integrated into the NANOBEM toolbox. The toolbox also provides help pages that can be accessed in the MATLAB help browser and contains a number of useful demo programs.

## 2 Theory

In our theoretical approach, we consider nanoparticles immersed in a fluid, which are illuminated by electromagnetic fields, typically those of a focused laser beam. The optical response of a nanoparticle is described in terms of the transition or T-matrix,^21,22^ which connects the multipole coefficients for the incoming fields *q*_inc_ to the coefficients for the scattered fields *a*_sca_ through
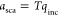
1This expression is generally valid for any scattering problem in linear response and requires the knowledge of the T-matrix that can be either computed analytically or semianalytically for simple shapes or numerically for more complicated nanoparticle geometries. In this work, we will be solely interested in spherical scatterers, where the T-matrix elements are given by the usual Mie coefficients,^14,23^ and ellipsoidal scatterers, where the T-matrix elements can be obtained in the form of one-dimensional integrals,^24,25^ but the toolbox can in principle cope with any type of particle geometries. From the incoming and scattered fields, obtained through eq 1, one can compute all optical quantities of interest, such as scattering cross sections, the power radiated into some solid angle, or optical forces and torques.^3,26^

When the nanoparticle propagates in the presence of optical and drag forces in the fluid, Newton’s equation of motion can be expressed as
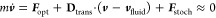
2Here, *m* is the mass of the particle, which might include the added mass due to the fluid,^27^***v*** is the particle velocity and ***v***_fluid_ the fluid velocity, which is usually set to zero for optical tweezers, ***F***_opt_ is the optical force, **D**_trans_ is the Stokes drag tensor for translation, and ***F***_stoch_ are the stochastic forces which are needed to counterbalance the drag.^3,12^ They are related to the drag tensor through the fluctuation dissipation theorem, as described in more detail in refs (3 and 28) and Appendix A. We additionally assume that the momentum relaxation time is so short that we can approximately set ***v*˙** ≈ 0,^27^ which corresponds to a particle flow in the regime of small Reynolds numbers.^29^ For a spherical particle, the drag tensor has the usual form **D**_trans_ = −6πη*R*_hyd_**I**, where η is the fluid viscosity, *R*_hyd_ the hydrodynamic radius, and **I** the unit tensor.

For the propagation of a spherical particle in a fluid, the set of eqs 1 and 2 has to be solved in parallel. Note that the incoming solution *q*_inc_(***r***) depends on the fields at the position ***r***(*t*) of the particle, and in turn, also the scattering coefficients *a*_sca_(***r***) and the optical force ***F***_tot_(***r***) depend on ***r***. For a nonspherical particle, we have to additionally account for the rotational motion, and the equation of motion for the angular velocity ω can be expressed in a form similar to eq 2
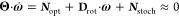
3Here, **Θ** is the tensor for the moment of inertia of the nonspherical particle, ***N***_opt_ is the optical torque,^3^**D**_rot_ is Stokes’ drag tensor for rotation, and ***N***_stoch_ is the stochastic torque which is needed to counterbalance the drag. Again we consider the regime of low Reynolds numbers where the inertial forces, described by the left-hand side of eq 3, can be neglected. In this paper, we only consider the case that the drag tensors are diagonal and, correspondingly, neglect direct couplings between translation and rotation, although such couplings are implemented in the toolbox as discussed in the Supporting Information.

Equations 1–3 form the basis of our simulation approach. They are identical to the working equations of tweezer simulations.^3,12^ In OF2i, however, the nanoparticles are trapped in the transverse directions only and move freely in the laser propagation direction. For this reason, it is often not possible to compute *q*_inc_ once at the trap center and translate it in a second step to the particle position. Rather we have to constantly compute *q*_inc_ at each nanoparticle position, which leads to a slightly different simulation approach. Nevertheless, optical tweezers and OF2i simulations are similar and can be approached with a unified simulation platform, as will be discussed in the next section.

## 3 Unified Simulation Platform

**Figure 1:**
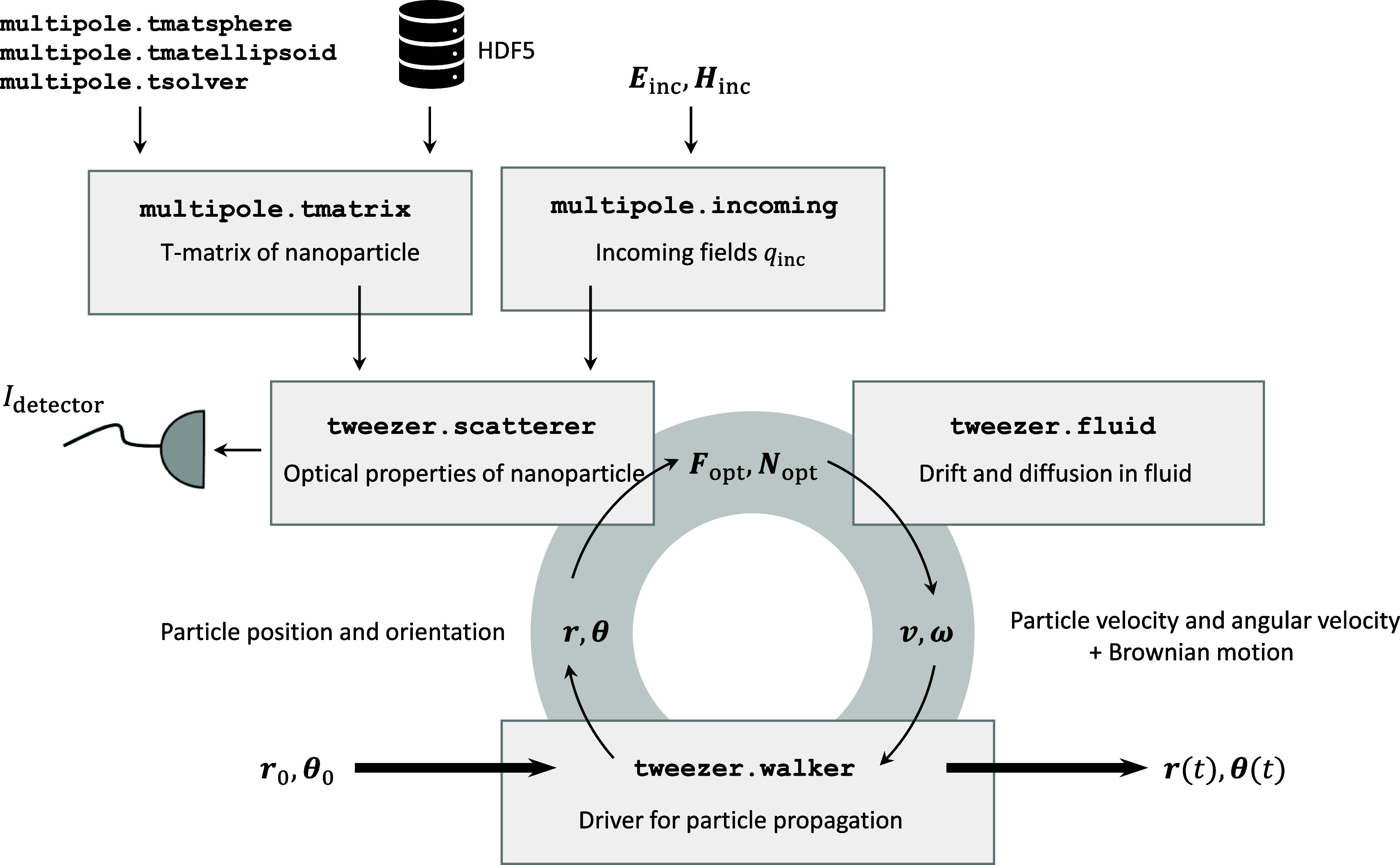
Schematics of the unified simulation platform. The main simulation loop consists of the tweezer.scatterer and tweezer.fluid classes, which define the optical properties of the nanoparticle and the solution of Newton’s eqs 2 and 3 in the presence of the fluid. tweezer.walker is a driver class that propagates the particles in time or space. The scatterer class is initialized with a T-matrix, which can be computed using either toolbox functions or by loading them from an external HDF5 file, and a functor multipole.incoming that computes the incoming field coefficients. For details, see the text.

We set out to discuss the ingredients of our unified simulation platform, which has been implemented as an add-on to the NANOBEM toolbox. The various ingredients are implemented as class objects that can be combined flexibly for various simulation setups. In our discussion, we only describe the general design criteria of the objects, see also Figure 1, and refer for technical details to the Supporting Information.

### 3.1 T-Matrix

In the toolbox, multipole.tmatrix objects store the transition matrices. The objects additionally hold the material properties of the embedding medium. We provide functions for fast evaluation of T-matrices for spherical and ellipsoidal particles and use for the latter the approach of refs (24 and 25), however, without the refined integration scheme. For particles with more complicated geometries, the NANOBEM toolbox provides a generic T-matrix solver suitable for particles composed of homogeneous materials that are separated by abrupt boundaries, such as coated particles.

As stated in the introduction, the NANOBEM toolbox is part of the T-matrix initiative of Carsten Rockstuhl and co-workers^20^ that suggests a unified storage format for T-matrices. This allows the transfer of T-matrices computed with different Maxwell solvers such as COMSOL or MEEP. In the future, it is also planned to set up a T-matrix database, which would allow the import of various precomputed T-matrices in a HDF5-format into our tweezer simulations. In our toolbox, we provide a number of functions for the manipulation of T-matrices, including rotation, translation, and the coupling of different T-matrices.

### 3.2 Incoming Fields

The multipole.incoming class allows computation of the multipole coefficients *q*_inc_, which are often also referred to as beam-shape coefficients, for user-defined incoming fields ***E***_inc_ and ***H***_inc_. The computation is based on eq(9.123) of ref (30). In ref (18), we have discussed the toolbox features for the simulation of focused laser fields, which are fully compatible with the multipole.incoming class. If needed, these fields can also account for aberration due to optical path differences^31^ as occasionally needed for oil immersion objectives. In the context of OF2i with its weakly focused laser beams, it is usually advantageous to use analytic expressions obtained in the paraxial approximation,^32^ as they allow us to considerably speed up the simulations.

### 3.3 Scatterer

The optical properties of the nanoparticles are accounted for by tweezer.scatterer objects, which hold the T-matrix and a functor for the evaluation of the incoming multipole coefficients *q*_inc_, typically in the form of a multipole.incoming object. The main task of the scatterer object is to provide the optical forces and torques for given positions and orientations of the particle,^3,26^ see also the Supporting Information for the detailed expressions used in our approach. In some cases, one also directly needs the scattering coefficients *a*_sca_, for instance, for computing the scattering cross section or the power radiated into the solid angle of a detector.

Occasionally, the computation of incoming coefficients *q*_inc_ and the optical forces can be time-consuming. For such situations, we provide grid objects tweezer.griddedScatterer, where forces, torques, and other user-defined quantities such as scattering cross sections are first computed on a grid and are then interpolated. See the Supporting Information for a graphical representation and further details. Gridding allows for fast simulations of up to a million particles, as sometimes needed in the context of OF2i. The computational grids are either in the radial and azimuthal directions for a given propagation distance, *z*, or fully three-dimensional. We perform a Fourier transform along the azimuthal direction and assume that for the quantities of interest, only a small number of angular degrees are needed.

For nonspherical particles, an additional interpolation is needed for the particle orientation. We have only implemented an interpolation for scatterers with a rotation symmetry along the *z* axis, and we use an expansion in terms of spherical harmonics. Typically, the corresponding simulations are significantly slower; nevertheless, gridding for nonspherical particles might be helpful in several cases. For particles without any symmetry, gridding becomes cumbersome and one should consider alternative approaches, such as neural networks that have been recently investigated in a related context.^33^

### 3.4 Drag Force and Diffusion

We provide the class tweezers.fluidsphere for spherical scatterers and the evaluation of eq 2. For ellipsoidal scatterers or those with rotational symmetry around the *z* axis, the class tweezer.fluidpol can be used for the solution of eqs 2 and 3. For particles with arbitrary geometries, we provide the class tweezer.fluidparticle. These classes store the viscosity η and temperature *T*, as needed for the simulation of Brownian motion, and are initialized with the drag tensors. Upon neglect of Brownian motion, one can compute for given optical forces and torques the drift velocities ***v*** and angular velocities **ω** and solve the particle trajectories using any ode-solver. When considering Brownian motion, we provide functions that propagate the particles over a given time step Δ*t* or propagation distance Δ*z*, considering drift and diffusion, the latter being implemented in the form of random Gaussian noise as described in more detail in ref (3) and Appendix A.

### 3.5 Walkers

The classes described above form the heart of OF2i and tweezer simulations. We provide two additional wrapper classes, tweezer.walkersphere and tweezer.walkerparticle, that can be used for a user-friendly solution of eqs 1–3. However, depending on the problem under consideration, it might be useful to solve the equations within a more open loop structure, for instance, when evaluating additional quantities after each time or propagation step, such as the scattered power.

Let us briefly comment on the accuracy and efficiency of our computational approach. Our BEM implementation is based on a Galerkin scheme^14^ that is known to converge to the exact solutions for sufficiently small mesh sizes.^34^ This is demonstrated in the example of the T-matrix for a spherical scatterer in the Supporting Information. For the spherical and ellipsoidal particles considered in this paper, the T-matrices can be evaluated analytically or semianalytically, and accuracy or speed is not an issue. Regarding the optical forces and torques, we use semianalytical expressions and show in the Supporting Information for a few selected particles and incoming fields that the results obtained with the NANOBEM toolbox agree perfectly well with those of the OTT toolbox,^13^ which is often employed in the field of optical tweezers, thus validating our simulation approach. With the exception of the force and torque gridding described above, all our simulations perform fast, and we have thus refrained from benchmarking our approach against other codes.

## 4 Results and Discussion

In this section, we present selected results for optical tweezer and OF2i simulations. Throughout, we consider polystyrene nanoparticles immersed in water, which are excited by incoming laser fields. The pertinent simulation parameters are listed in Table 1. More information about the simulation programs can be found in Supporting Information and the help pages of the toolbox.

**Table 1:** Parameters Used in Our Tweezer and OF2i Simulations

description	symbol	value
refractive index water	*n*	1.33
refractive index polystyrene	*n*_*p*_	1.59
viscosity water at 293 K	η	9.544 × 10^–4^ Pa s
temperature	*T*	293 K
fluid velocity in OF2i	*v*_fluid_	0.3 mm s^–1^
laser wavelength for the tweezer (Gauss)	λ	520 nm
laser power	*P*	1 mW
laser polarization	**ϵ**	*x*-polarized
numerical aperture for the focusing lens	NA	1.0
laser wavelength for OF2i (Laguerre–Gauss)	λ	532 nm
radial index and topological charge	(*n*, *m*)	(0,2)
laser power	*P*	1.65 W
laser polarization	**ϵ**	*y*-polarized
laser width in the focus plane^32^	*w*_0_	4.78 μm

### 4.1 Optical Tweezers and Spherical Particles

**Figure 2:**
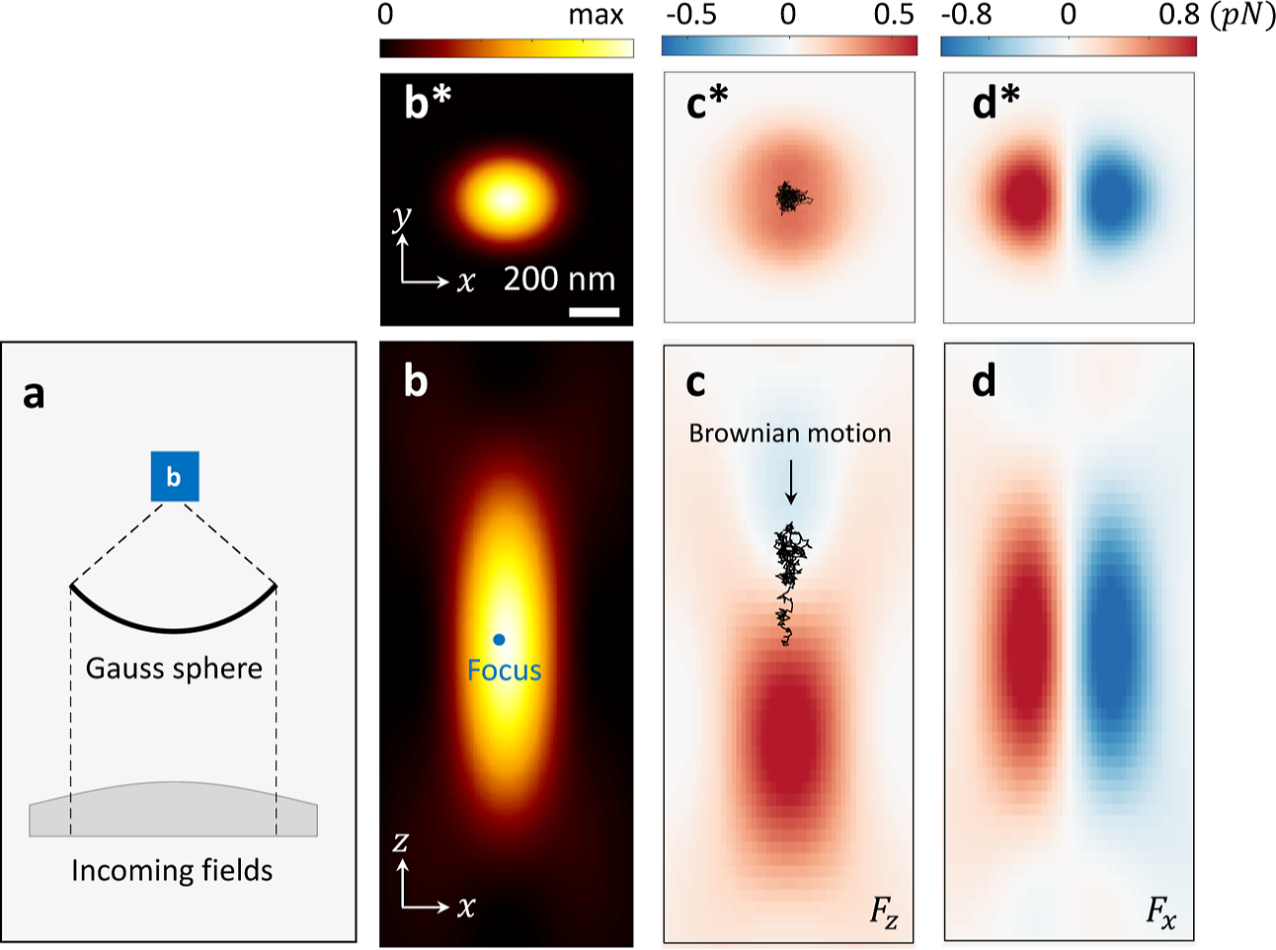
Tweezer simulations. (a) We consider an incoming Gaussian laser field that becomes focused by a lens, using the optics.lensfocus object provided by the toolbox for generic incoming fields.^18^ Panels (b) and (b*) report the light intensity in the *xz* and *xy* planes, respectively. The force exerted on a polystyrene nanosphere with a diameter of 400 nm is shown in panels (c,c*) for *F*_*z*_ and (d,d*) for *F*_*x*_ in pico-Newton (see colorbars on top of the panels). In panels (c,c*), we also show the trajectories of Brownian motion, as obtained with the tweezer.walkersphere object provided by the toolbox. In the simulations, the particle starts at the focus point.

We start our discussion with optical tweezer simulations. It should be emphasized that our software has not been developed and optimized for tweezers; nevertheless, our toolbox functions are sufficiently generic and versatile to easily allow also for such simulations. Figure 2 shows a typical simulation setup, where an incoming Gaussian laser field becomes focused. In the simulations, this is accomplished by optics.lensfocus objects, previously described in ref (18). The corresponding intensity maps are shown in panels (b,b*). Because of the tight focusing, particles can become trapped in regions with high field intensities. The optical forces for a polystyrene nanosphere with a diameter of 400 nm are shown in panels (c,d). The trap minimum is slightly above the focus point; here, the scattering and gradient force compensate each other. The black curves in panels (c,c*) show the trajectory for a single nanosphere in the presence of Brownian motion.

With the NANOBEM toolbox, tweezer simulations can be usually set up easily with typical runtimes of seconds to minutes on a normal desktop computer. The probably slowest part is the evaluation of the incoming fields and inhomogeneities *q*_inc_, see eq 1, using the plane-wave decomposition objects optics.decomposition provided by the toolbox. These objects have been mainly designed for versatility and not speed and exploit, for instance, no rotational symmetries. Correspondingly, they can be used for slight off-axis illuminations or for field propagation through stratified media, as is often the case for the consideration of coverslips. In general, more efficient field evaluations will allow us to speed up the simulations. In the toolbox, we also provide the possibility to compute *q*_inc_ at a specific point, such as the trap minimum, and to shift the multipole coefficients of *q*_inc_ by means of translation matrices,^3,35^ a procedure conveniently employed for optical tweezers. Alternatively, one can also initially compute the optical forces on an equidistant grid and interpolate them later in trajectory simulations. We have tested all methods and have found that translation works best for small spheres, where the cutoff in angular degrees can be chosen low, whereas gridding and tabulation work best for larger particles.

### 4.2 OF2i and Spherical Particles

We next discuss OF2i simulations. The basic mechanism underlying OF2i has been presented in refs (8 and 9). In short, it is a nanoparticle characterization scheme with single-particle sensitivity and high throughput. The particles to be analyzed are immersed in a fluid, typically water, and are pumped through a microfluidic channel alongside a weakly focused laser beam, which can trap particles in the transverse directions *x* and *y*. In the propagation direction *z*, the particles experience scattering forces *F*_*z*_ due to the focused laser beam and correspondingly move faster. By measuring the velocity enhancements of the individual particles, one obtains particle sizes and in turn particle size distributions.

**Figure 3:**
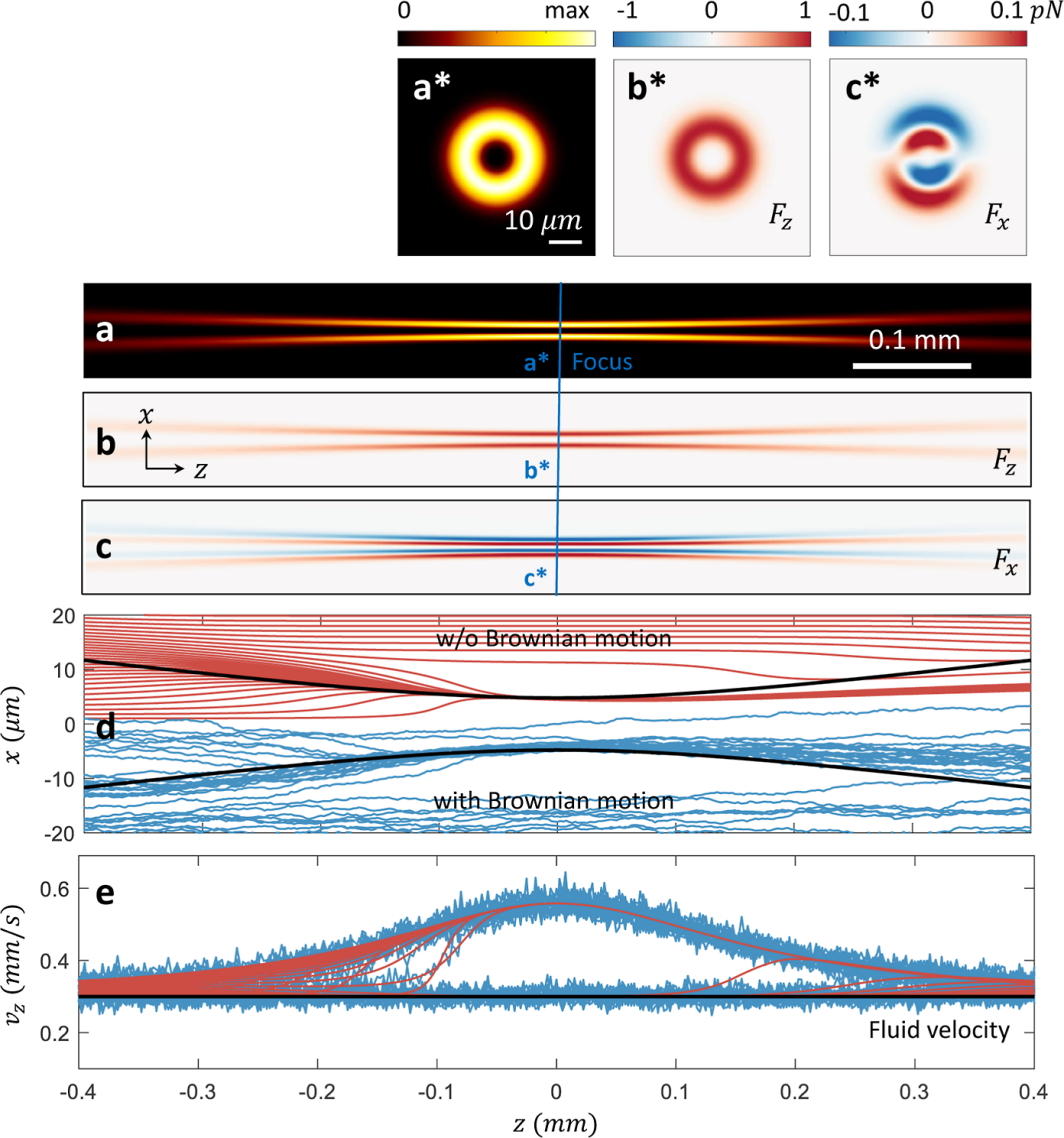
Simulation of optofluidic force induction (OF2i). A weakly focused Laguerre–Gauss beam with a topological charge of *m* = 2 propagates in the *z* direction. Panel (a) shows the intensity profile in the *xz* plane and panel (a*) in the *xy* plane at *z* = 0 (focus plane). The optical forces are shown in (b,b*) for *F*_*z*_ and (c,c*) for *F*_*x*_ for a polystyrene nanosphere with a diameter of 400 nm. The trajectories of nanospheres starting at different initial positions (*z*_0_ = – 1 mm) are shown in panel (d) for simulations that ignore Brownian motion (top) and consider Brownian motion (bottom). One observes that spheres sufficiently close to the intensity maxima become trapped in the transverse direction and then continue to propagate along the intensity maxima. The corresponding velocities are shown in panel (e). Due to the scattering forces, the nanoparticles experience velocity enhancements, which are used in experiment for the determination of nanoparticle sizes.^8−10^

Figure 3a shows the intensity profile of a weakly focused Laguerre–Gauss beam, which is conveniently employed in OF2i experiments. To minimize unwanted collisions in the focus region, the incoming laser has an optical angular momentum that results in the ring-like intensity profile shown in panel (a*). The optical forces for a polystyrene nanosphere with a diameter of 400 nm are shown in panels (b,b*) for the scattering force *F*_*z*_ and in panels (c,c*) for the trapping force *F*_*x*_.

Because in OF2i, the nanoparticles are not trapped in all three spatial dimensions but can move freely along *z*, the multipole coefficients *q*_inc_ need to be constantly computed along the particle trajectories, contrary to optical tweezers, where they can be updated through translation matrices. However, for the weakly focused laser beam, the fields can be computed in the paraxial approximation,^32^ which results in a fast computation of *q*_inc_. Below, we will also show that one can successfully employ the interpolation of tabulated data for fast force evaluations.

In Figure 3d, we show selected trajectories of the nanospheres for simulations that ignore (top) and include (bottom) Brownian motion. In both cases, we observe that the nanospheres become trapped in the transverse directions and continue to move along the intensity maxima of the Laguerre–Gauss beam. The resulting velocity enhancements due to the scattering forces *F*_*z*_ are reported in panel (e), showing a velocity increase from the fluid velocity of 0.3 mm s^–1^ to values of approximately 0.6 mm s^–1^ in the focus region. This velocity enhancement depends on the sphere size, since the velocities and optical forces increase with increasing particle size, which is used in OF2i for a determination of the particle sizes from the measurement of velocities.^8−10^

Owing to the trapping forces that increase with particle size, typically larger particles are trapped more easily and observed more frequently in OF2i measurements. Thus, when evaluating particle concentrations from OF2i, one needs to account for the trapping efficiencies. To this end, the NANOBEM toolbox is designed such that one can perform simulations with up to 1 million walkers with moderate runtimes of a few minutes. We here exploit that eq 2 can be solved for particles transported by the fluid as a function of propagation distance *z* rather than time. Initially, all walkers start in a region of small field intensities at different transverse positions *x* and *y* but the same propagation distance *z*. Next, within each update step, the walkers are propagated to a new common *z* value, using optical forces that are interpolated from tabulated data, which are computed for a few transverse positions and a single *z* value. This results in a massive speedup in comparison to the direct force evaluation for each individual walker. We additionally use a box with periodic boundary conditions in the transverse directions, such that walkers that move away from the focused laser beam and could no longer be observed in the OF2i imagining re-enter at the other side of the periodicity box and thus have a second chance for diffusing toward the laser. This simulation setup corresponds to the actual OF2i measurements where particles are distributed inside the relatively large capillary and constantly move toward and away from the laser, but only particles close to regions of sufficiently high laser intensity can be actually imaged.

**Figure 4:**
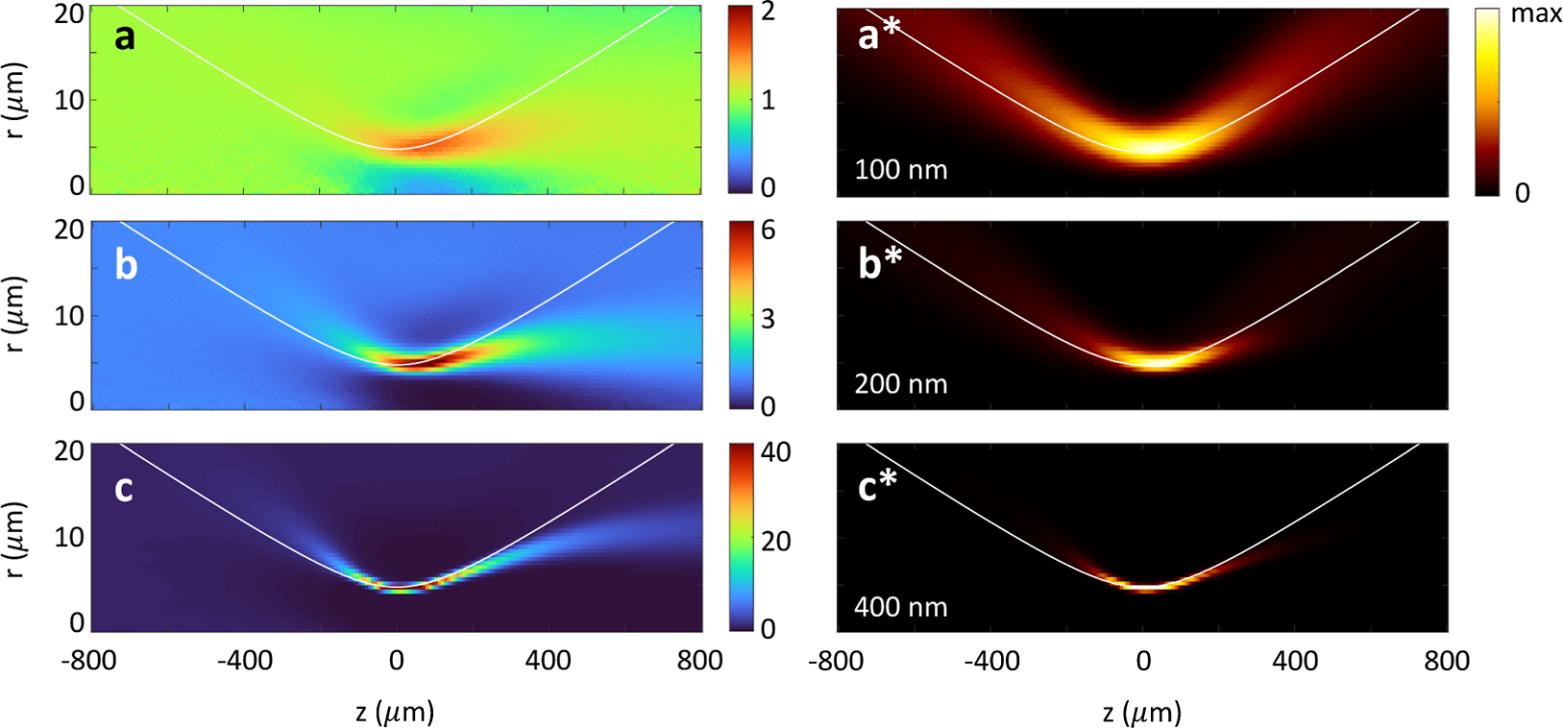
Brownian motion of nanospheres in OF2i. Panels (a–c) show the particle density at different radial and propagation distances *r* and *z*, respectively, and for different sphere diameters of (a) 100 nm, (b) 200 nm, and (c) 400 nm. Because of optical trapping, the density increases at the intensity maxima of the incoming laser beam, see colorbars. (a*–c*) Total scattered power, which is proportional to the product of particle density and laser intensity. In the simulations, we consider a million walkers. The white lines indicate the intensity maxima of the incoming laser.

Figure 4 shows simulation results for one million nanospheres with different diameters (see the inset and caption) that propagate through the OF2i setup in the presence of Brownian motion, as described above. Panels (a–c) report the particle density as a function of transverse and propagation distance *r* and *z*. Panels (a*–c*) show the total scattered power of all particles at different radial and propagation distances *r* and *z*. In our simulations, we first set up a radial grid with intervals [*r*_*i*_, *r*_*i*_ + Δ*r*] and count the number of particles *N*_*i*_ within each interval. The densities are then obtained by dividing *N*_*i*_ with the phase space volumes 2π*r*_*i*_Δ*r* and introducing an overall scaling factor that is chosen such that the density is for a homogeneous distribution. Thus, the density maps show the density enhancements due to the optical forces which trap the particles. For the smallest spheres with a diameter of 100 nm (panel a), the density enhancements are only visible in the focus region and bound to values below two. With increasing sphere diameter (panels b,c), the density enhancements dramatically increase in regions of high field intensities, where particles become trapped, and correspondingly, the density drops in neighbor regions where the particles have moved away. Panels (a*–c*) report the total scattered power as a function of *r* and *z*. For the smallest spheres (panel a*), the light scattering is almost proportional to the laser intensity at *r* and *z*, with an only moderate increase in the focus region due to the enhanced particle density there. Things change dramatically for larger spheres (panels b* and c*) where the intensity strongly increases in the focus region because of the more efficient trapping and the strongly increased particle density there.

A critical point in OF2i is the choice of the “active volume”,^8−10^ which accounts for the fact that larger particles are trapped more frequently. This has to be corrected for in the evaluation of the particle size distribution. Previously, we have computed this quantity under the assumption that Brownian motion can be neglected.^9^ In the following, we investigate the influence of Brownian motion and introduce a qualitative measure for the size-dependent trapping efficiency by comparing results of simulations where optical forces are either considered or artificially ignored. By computing the ratio between the simulation results in the focus plane, we obtain the scattering enhancement factor .  is close to one for small particles where trapping is weak; here, the particle density distribution is practically homogeneous, see Figure 4a.  increases for larger particles that are trapped more efficiently at the intensity maxima of the incoming laser and consequently scatter more light.

**Figure 5:**
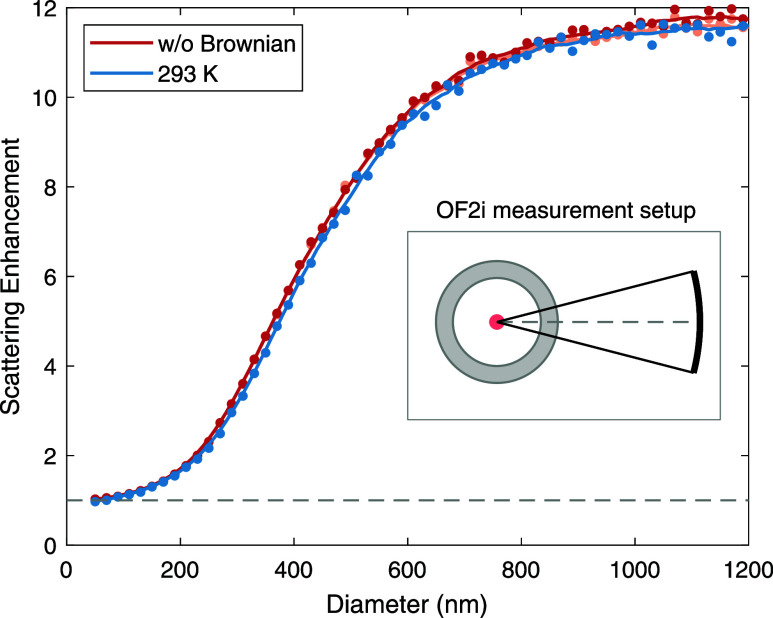
Scattering enhancement in OF2i. We show the ratio of the scattered power for simulations that either consider or neglect optical forces, as discussed in the text, and for different nanosphere diameters. We compare simulation results with and without consideration of Brownian motion and for the scattered power being integrated over the entire solid angle (dark lines) or the small angle shown in the inset (bright lines, indistinguishable).

In Figure 5, we compare simulations without and with Brownian motion at room temperature and for different sphere diameters. The solid lines are a guide to the eye for the simulation results (dotted line), which start to weakly oscillate at larger diameters indicating the onset of Mie resonances.^9^ With increasing sphere diameters, the scattering enhancement increases, which is in agreement with the results of Figure 4 showing an increased density and scattering in the vicinity of the intensity maxima of the incoming laser. We find that the scattering enhancement is very similar for simulations neglecting and including Brownian motion. In the presence of Brownian motion, the scattering enhancement even slightly decreases, which we attribute to processes where particles weakly trapped in low-intensity regions are pushed away by the random Brownian motion into regions of even lower intensity. Finally, we compare in the figure simulation results for the total scattered power (dark lines) to the scattered power radiated into a solid angle for a lens with a numerical aperture of NA = 0.25 (bright lines, indistinguishable), as shown in the inset of the figure. The optical axis of the imaging lens is oriented along *x*. The latter setup corresponds to the imaging geometry of the OF2i experiments. We observe that the consideration of a restricted solid angle has practically no impact on the scattering enhancement.

### 4.3 Nonspherical Particles

We conclude this section with a discussion about the tweezer and OF2i simulations of nonspherical particles. The toolbox provides a number of generic classes and functions for the computation of T-matrices and the drag tensors for particles of arbitrary shape, but we have only tested ellipsoidal particles so far, for which simplified expressions can be obtained.^3,24,25^

**Figure 6:**
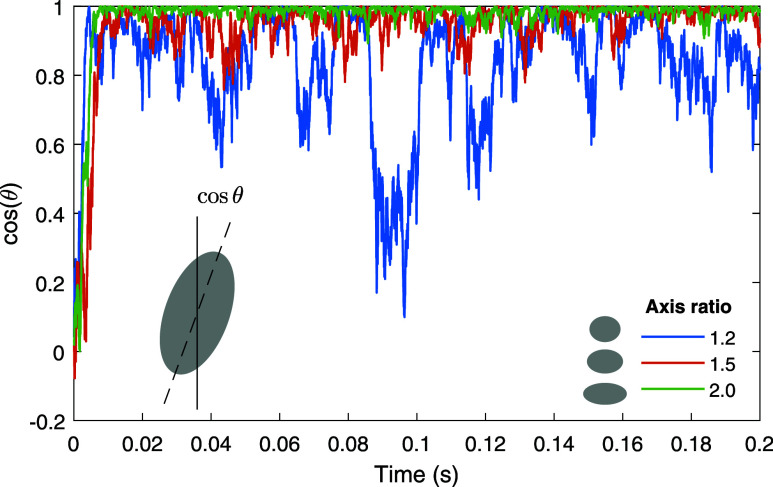
Orientation of ellipsoids in an optical tweezer. We consider prolate ellipsoids with a volume corresponding to a sphere with 400 nm diameter and with the axis ratios reported in the inset. We plot the cosine of the angle between the symmetry axis of the ellipsoid and the *z* axis of the focused laser, see Figure 2, as a function of time. With increasing axis ratio, the optical torque increases, which suppresses the Brownian rotation diffusion of the ellipsoids. Initially, the particle is at the focus point and has an orientation of θ = 0.

Figure 6 shows simulation results for prolate ellipsoids with the different axis ratios reported in the inset and for a volume corresponding to a sphere with a diameter of 400 nm. We show the cosine of the angle θ between the symmetry axis of the ellipsoid and the optical axis *z*, see Figure 2. Due to the optical torque exerted by the focused laser beam, the ellipsoids are preferentially aligned parallel to the *z* direction, and they exhibit rotational Brownian motion around the trapping direction. With increasing axis ratio, the optical torque increases and the Brownian fluctuations become suppressed.

**Figure 7:**
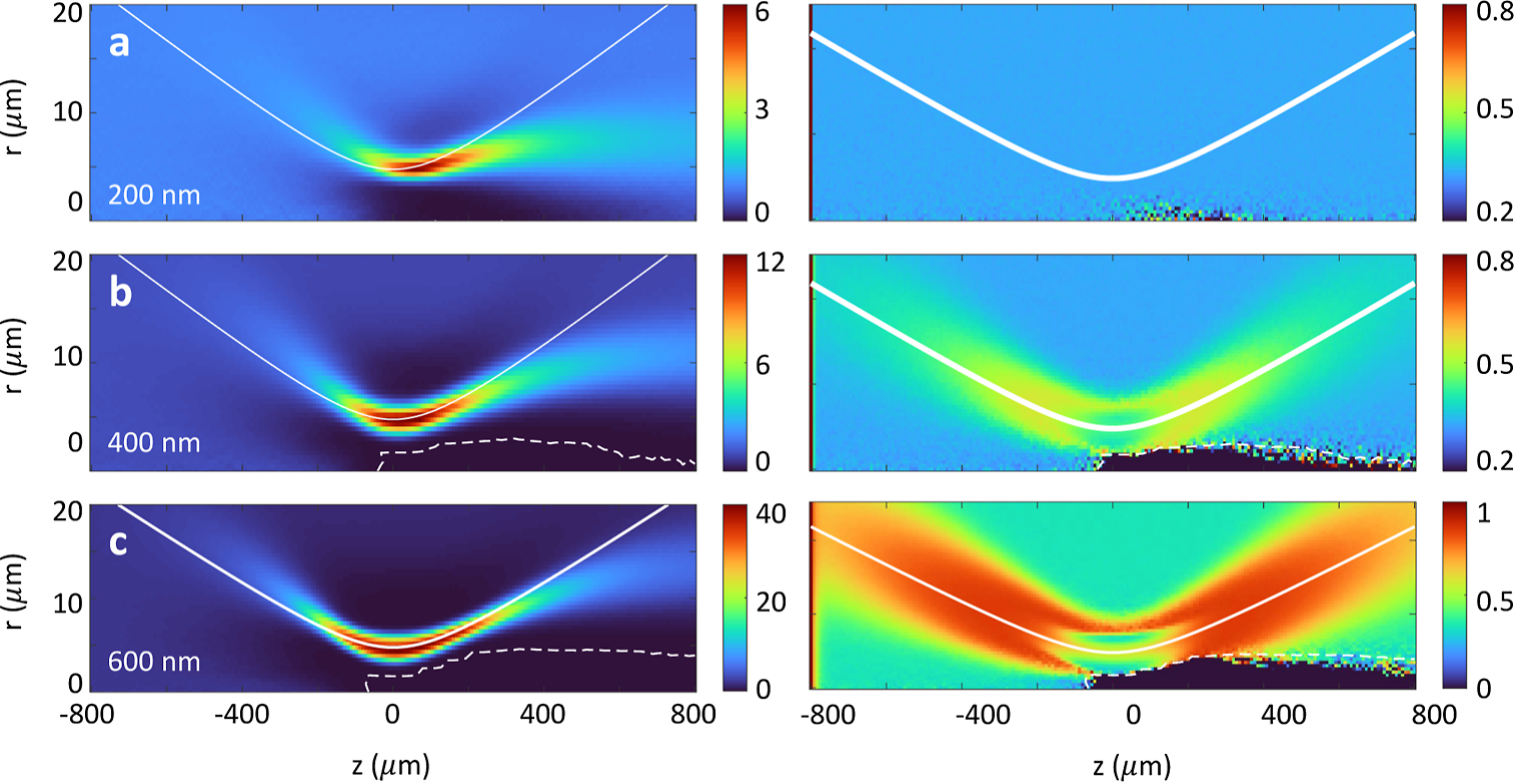
Brownian motion of nanoellipsoids in OF2i. We consider nanoellipsoids with an axis ratio of 1:2 and for a volume corresponding to the sphere volume for the diameters indicated in the panels. (a–c) Density enhancement similar to Figure 4a–c. (a*–c*) Mean value of orientation factor cos^2^θ indicating the preferential orientation of the ellipsoids. For small particles, the orientation is almost random with a small bias due to the different components of the rotational drag tensor **D**_rot_. With increasing particle size (panels b*,c*), the optical torque increases and the ellipsoids are preferentially oriented along the laser propagation direction *z*. Note that for small *r* values and propagation distances *z* > 0, no particles are detected (dashed lines, dark area). In the simulations, initially, all particles start at *z*_0_ = – 0.8 mm with arbitrary transverse positions and an orientation θ = 0.

Figure 7a–c reports the particle density enhancement of ellipsoidal particles with an axis ratio of 1:2 and for different particle sizes, see the inset. The results are similar to those for the spherical particles shown in Figure 4. In the simulations, we consider a million particles and perform interpolation of the optical forces and torques on tabulated data, using the tweezer.griddedScattererPol objects described in the Supporting Information. The corresponding simulations are slower by a factor of about four in comparison to spherical particles, with runtimes on the order of a few minutes. For the orientation of the ellipsoids, we have to be careful that because of symmetry the average value of cos θ is zero, since ellipsoids pointing “upwards”, cos θ = 1, are indistinguishable from ellipsoids pointing “downwards”, cos θ = – 1. In Figure 6, we choose the angle θ such that at long times, cos θ fluctuates around one. Panels (a*–c*) in Figure 7 plot the expectation value of cos^2^θ. For the smallest ellipsoid shown in panel (a*), the orientation is almost the same for all positions; here, the optical torque is too weak for aligning the particles. With increasing size, panels (b*,c*), we observe a preferential alignment in the *z* direction close to the intensity maxima of the incoming laser. Note that in all simulations, the initial condition at *z* = −800 μm is such that the particles point along *z* and that for small *r* values and propagation distances *z* > 0, no particles are detected (dashed lines, dark area).

**Figure 8:**
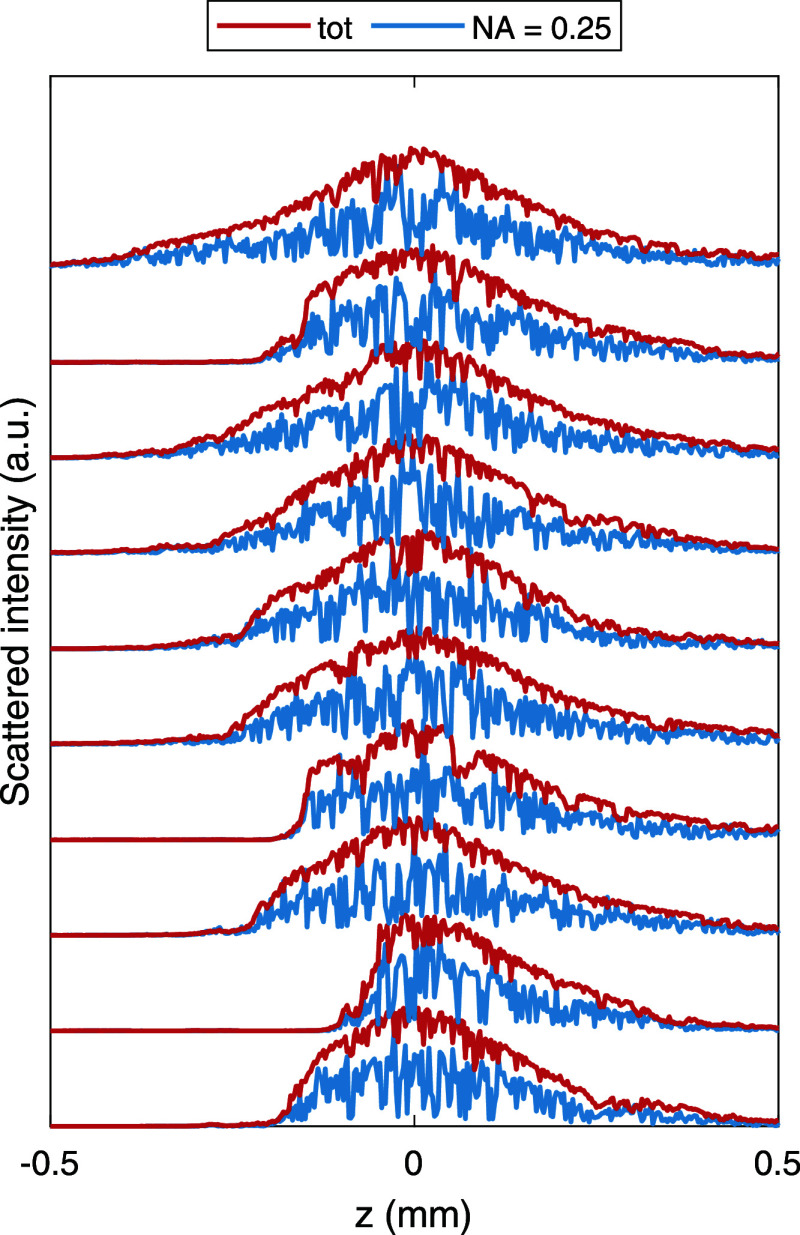
Scattered power of ellipsoids (axis ratio 1:2, equivalent diameter of 400 nm) in OF2i for different particles. The different curves are offset for better visibility. We compare the results of the total scattered power (red lines) to those where the scattered light is detected in a limited solid angle (blue lines), corresponding to the optical setup shown in the inset of Figure 5.

Finally, in Figure 8, we show the scattering power for selected trajectories of the ellipsoids. We compare the total scattered power (red lines) to the power radiated into the solid angle of the imaging lens, shown in the inset of Figure 5. The total scattered power fluctuates because of the translational Brownian motion, where the particles hop on and off the intensity maxima. In contrast, for the reduced solid angle, we mainly observe rotational Brownian motion. The light scattering of the ellipsoid is strongly peaked along the symmetry axis. Thus, the scattering into the directions of the detector depends on the particle orientation, and thus, rotational Brownian motion translates to pronounced fluctuations in the detector signal.

## 5 Summary and Outlook

To summarize, we have presented a unified simulation platform for optical tweezers and optofluidic force induction. In our approach, we consider optical and fluidic forces and characterize the optical response in terms of T-matrices. Newton’s equations of motion are solved under the assumption of low Reynolds numbers. The methodology has been implemented in the NANOBEM toolbox, and we provide the software in the Supporting Information together with a detailed discussion of the novel toolbox features. Although we have been concerned only with spherical and ellipsoidal particles, it is relatively straightforward to run simulations for other particle geometries.

The NANOBEM toolbox is a generic Maxwell solver based on the boundary element method. One can compute T-matrices for arbitrary nanoparticle geometries. The tweezer and optical force features presented in this paper have been included as an add-on to the toolbox. Together with the various optics features recently added to the toolbox, which include the computation of focused laser beams and imaging, we think that our software constitutes a flexible toolkit for various applications.

## Appendix A

### Brownian Motion

In this appendix, we briefly discuss our implementation of Brownian motion. For a spherical particle, Newton’s equation of motion for a particle subject to optical and fluidic forces is given by eq 2, which we repeat here for clarity

4

In the absence of ***F***_stoch_, we obtain from this equation the particle velocity

5

When considering Brownian motion, the Stokes drag and the stochastic force ***F***_stoch_ are connected through the fluctuation dissipation theorem.^3^ A convenient way to account for Brownian motion is by introducing Gaussian random variable *W* with a unit variance. Within a short time interval δ*t*, the sphere position then changes according to (eq 18) of ref (12)

6

A similar procedure can be pursued in the case of nonspherical particles. We assume that both the translational and rotational drag tensors **D**_trans_ and **D**_rot_ are diagonal, as is the case for particles with a polar symmetry. Let **R**(*t*) be a matrix that rotates the particle at time *t* to the laboratory frame. In the absence of Brownian motion, the particle velocity and angular velocity can be obtained from eqs 2 and 3 according to

7

We have used in the bracket terms **R**(*t*) to rotate the drag tensors to the laboratory frame. Because both of them are diagonal in the particle frame, we finally add translational and rotational Brownian motion in the particle frame and then transform to the laboratory frame via

8

We thus need six Gaussian random variables to update the particle position and orientation. The time step δ*t* should be chosen sufficiently small, such that neither the optical force, torque, nor particle orientation changes significantly during the update step.

## Supporting Information Available

Simulation software and demo programs for selected optofluidix simulations (ZIP)
User guide with installation details for the MATLAB toolbox for optofluidic simulations, comparison of results of our toolbox with those of the optical tweezer toolbox (OTT), and methodology underlying the computation of optical forces and torques (PDF)
