# Supplementary material for: Unified Simulation Platform for Optical Tweezers and Optofluidic Force Induction
Source: ACS Photonics. 2025 Mar 22;12(4):2242–51. doi: 10.1021/acsphotonics.5c00254 (PMC12007102; doi:10.1021/acsphotonics.5c00254)
Supplement: Supplementary file 2 — ph5c00254_si_002.pdf [file ph5c00254_si_002.pdf]

Unified simulation platform for optical tweezers and optofluidic force induction: Supplementary Information

Ulrich Hohenester,[1] Marko Šimić,[1] Raphael Hauer,[1,2] Lorenz Huber,[1] and Christian Hill[2,3]

[1]*Institute of Physics, University of Graz,*
*Universitätsplatz 5, 8010 Graz, Austria**
[2]*Brave Analytics GmbH, Austria*
[3]*Gottfried Schatz Research Center, Division of Biophysics,*
Medical University of Graz, Neue Stiftingtalstraße 2, 8010 Graz, Austria

In this Supplementary Information we present and discuss the main features of the `+tweezer` folder containing the additional classes required for the simulation of optical tweezers and optofluiduc force induction. The software is integrated as an add-on into the NANOBEM toolbox. We also show selected results to demonstrate the accuracy of our simulation software, and provide the equations for computing optical forces and torques.

28 pages S1–S28
7 figures S1–S7
0 tables

* E-mail `ulrich.hohenester@uni-graz.at`.

CONTENTS

S1. Short description of NANOBEM toolbox S2
S2. Validation of toolbox S17
S3. Theory S22
References ??

S1. SHORT DESCRIPTION OF NANOBEM TOOLBOX

A. Getting started

In this section, we briefly describe how to set up the toolbox and how to run simulations for optical tweezers and optofluiduc force induction (OF2i). To install the toolbox, one must add the path of the main directory `nanobemdir` of the NANOBEM toolbox as well as the paths of all subdirectories to the MATLAB search path. This can be done, for instance, through

```
>> addpath(genpath(nanobemdir))
```

To set up the help pages, one must once change to the main directory of the NANOBEM toolbox and run `makehelp`

```
>> cd nanobemdir
>> makehelp
```

Once this is done, the help pages, which provide detailed information about the toolbox, are available in the MATLAB help browser. See also [1–3] for further details. In the NANOBEM toolbox provided in the Supporting Information we have included the `+tweezer` folder into the `addons` directory of the toolbox. The command `addpath(genpath(nanobemdir))` then automatically makes all toolbox and `+tweezer` functions available.

The demo programs accompanying this paper are stored in the `Demo/tweezer` folder and can be directly accessed over the help pages. To run the demo programs, one must change within the MATLAB environment to this folder and execute the files.

B. A few simple examples

In this section we discuss a few simple examples for setting up simulations for optical tweezers and OF2i. The corresponding programs `demotweezer01.m` and `demowalkersph01.m`

can be found in the help pages of the toolbox. Typical simulations start by defining the materials of the nanoparticle and the embedding medium, and the wavenumber of the incoming excitation.

```
1  mat1 = Material( 1.33 ^ 2, 1 );   %  material property of water
2  mat2 = Material( 1.59 ^ 2, 1 );   %  material property of polystyrene
3  k0 = 2 * pi / 520;                %  wavenumber of light in vacuum
```

In lines 1,2 we define the material properties of water and polystyrene with constant refractive indices of 1.33 and 1.59, respectively. It is also possible to define dispersive material parameters, as described in [1] and the help pages. In line 3 we define the wavenumber of light in vacuum for the incoming excitation. Next, we define the T-matrix for a nanosphere and the Stokes drag for a sphere.

```
4  diameter = 400;     %  diameter of sphere
5  tmat = multipole.tmatsphere( mat2, mat1, diameter, k0 );  %  T-matrix of sphere
6  fluid = tweezer.fluidsphere( diameter );                  %  fluidic forces
```

From here on the simulation designs somewhat differ for optical tweezer an OF2i simulation simulations.

Optical tweezer simulation for nanosphere

In an optical tweezer simulation we next define the incoming laser excitation.

```
7  NA = 1.0;
8  lens = optics.lensfocus( mat1, k0, NA );  %  focus lens
9  e = 2 * normpdf( lens.rho, 0, 1 );
10 e = e( : ) * [ 1, 0, 0 ];              %  incoming fields
11 foc = eval( lens, e );                 %  planewave decomposition of focal fields
```

In line 8 we define a focusing lens with a numerical aperture `NA=1.0`, as described in more detail in [3]. The electric fileld impinging on the focus lens is provided in lines 9,10, and finally we compute the focus fields in line 11. `foc` is an object of type `optics.decompose` [3].

The `tweezer.scatterer` object to be defined next plays a central role in our simulations, see also Fig. 1 of the main text. For its definition we need a functor that allows evaluating the coefficients q_{inc} of the incoming excitation.

```
12 fun = @( pos, k0 ) fields( foc, Point( mat1, 1, pos ) );
13 qinc = multipole.incoming( mat1, k0, fun, 'lmax', tmat.lmax, 'diameter', diameter );
14 scatterer = tweezer.scatterer( tmat, qinc );
```

In line 12 we define an inline function that computes the electromagnetic fields `e,h` at the positions `pos`. The coefficients $q_{\rm inc}$ are computed using an object of type `multipole.incoming`. `lmax` is the maximal degree for the multipole expansion of the incoming fields, which should be identical to that of the T-matrix, the `diameter` parameter is discussed in more length in the help pages and below. Finally, in line 14 we set up the scatterer object that allows computing the optical forces and scattering properties of the T-matrix object.

We are now in the position to propagate the nanosphere using the forces exerted by the focused laser beam and the fluid.

```
15 pos = [ 0, 0, 0 ];  %  initial position of nanosphere
16 dt = 3e-5;          %  time step
17 for it = 1 : 1000
18   fopt = optforce( scatterer, pos );
19   pos = browniant( fluid, pos, fopt, dt );  %  update position of nanosphere
20 end
```

In lines 15,16 we set up the initial position of the nanosphere and define the time step. In a loop structure we then compute the optical forces (line 18) and update the nanosphere position in time using the `browniant` function of the `fluid` object defined in line 6. Instead of the loop we could have also introduced a `tweezer.walkersphere` object, as will be discussed next at the example of OF2i simulation.

OF2i simulation for nanosphere

The simulation program for OF2i has to be set up only slightly different. First, we add the constant fluid velocity `vel` to the `fluid` object and define the incoming fields of a weakly focused Laguerre-Gauss beam in the paraxial approximation [4].

```
fluid.vel = 0.3e-3;    %  fluid velocity in (m/s)

field = laguerregauss( mat1 );  %  weakly focused Laguerre-Gauss beam
field.foc = 50.8e6;             %  focus length
field.w0  = 1.8e6;              %  width of incoming beam
```

```
12 field.nLG = 0;
13 field.mLG = 2;                   %  topological charge
14 field.pow = 1.65;                %  laser power
15 field.pol = [ 0, 1, 0 ];         %  laser polarization
16
17 fun = @( pos, k0 ) paraxial( field, pos, k0 );  %  evaluation function
18 qinc = multipole.incoming( mat1, k0, fun, 'lmax', tmat.lmax, 'diameter', diameter );
19 scatterer = tweezer.scatterer( tmat, qinc );
```

For a detailed discussion of the laser parameters see [4]. We can now set up simulations for several nanoparticles, which we refer to as "walkers", that start at different initial positions.

```
20 x = linspace( 0, 30e3, 21 );
21 zout = 1000e3 * linspace( -1, 1, 201 );
22 pos = x( : ) * [ 1, 0, 0 ] + [ 0, 0, min( zout ) ];
23 walker = tweezer.walkersphere( scatterer, fluid, pos );   %  set up walker object
```

In lines 20–21 we define the initial positions `pos` that start at $z = -1$ mm and at different transverse positions x. Note that the toolbox uses length units of nanometers. In line 23 we set up an object of type `tweezer.walkersphere` that can be used to propagate the walkers through the OF2i setup.

```
24 [ wout, tout ] = propagatez( walker, zout );  %  propagate walkers w/o Brownian motion
25 posout = cat( 3, wout.pos ) * 1e-6;  %  walker positions (mm)
26 velout = cat( 3, wout.vel ) * 1e-6;  %  walker velocities (mm/s)
```

In lines 25,26 we store the output walker positions and velocities in a single array of size `n`×`3`×`nz`, where `n` is the number of walkers and `nz` the number of propagation distances `zout`. The function in line 24 computes the particle trajectories without Brownian motion. If we want to include Brownian motion in our simulations, we have to replace line 24 with

```
[ wout, tout ] = brownianz( walker, zout );  %  propagate walkers with Brownian motion
```

Below and in the help pages we discuss how to control the simulations through additional property pairs passed to the various class objects and functions.

Optical tweezer simulation for nanoellipsoid

The simulations have to be adapted only moderately for ellipsoidal nanoparticles. In the following we only discuss optical tweezer simulations, but things are very similar for OF2i

simulations. The line numbering we are referring to below corresponds to the case of the spherical particle in the optical tweezer. Lines 4–6 are replaced with

```
4 [ diameter, ratio ] = deal( 300, 2 );
5 tmat = multipole.tmatellipsoid( mat2, mat1, diameter, ratio, k0, 'lmax', 10 );
6 fluid = tweezer.fluidellipsoid( diameter, ratio );
```

In lines 4–6 we set up the T-matrix and the Stokes drag for an ellipsoidal particle with a diameter of 300 nm and an axis ratio of 1:2. The definition of the incoming fields (lines 7–11) and the scatterer object (lines 12–14) remain identical, but lines 13,14 must be re-evaluated for the T-matrix of the ellipsoid.

For the main loop of the simulations, we must additionally define a rotation matrix `rot`, which actively rotates the ellipsoid to the laboratory frame. For a single particle `pos` is an array of size `1`×`3` and `rot` an array of size `3`×`3`, for multiple particlses `pos` is an array of size `n`×`3` and `rot` an array of size `3`×`3`×`n`. In the simulation for a single particle, lines 15–20 of the main optical tweezer loop must be replaced with

```
15 pos = [ 0, 0, 0 ];  %  initial position of nanosphere
16 rot = eye( 3 );     %  initial rotation matrix
17 dt = 3e-5;          %  time step
18 for it = 1 : nt
19   [ fopt, nopt ] = optforce( scatterer, pos, rot );  %  optical force and torque
20   [ pos, rot ] = browniant( fluid, pos, rot, fopt, nopt, dt );
21 end
```

Other demo programs

The help pages of the toolbox provide a number of additional demo programs. We recommend users to first work through the demo programs and to consult the detailed help pages only at a later stage.

C. Tweezer classes and functions

The following classes and functions of the NANOBEM toolbox are needed for the simulation of optical and fluidic forces.

```
+tweezer.fluidsphere      %  Stokes drag and diffusion for sphere in fluid
+tweezer.fluidellipsoid   %  Stokes drag and diffusion for ellipsoid in fluid
```

```
+tweezer.fluidpol         %  Stokes drag and diffusion for particle with polar symmetry
+tweezer.fluidparticle    %  Stokes drag and diffusion for particle w/o symmetry
+tweezer.dragellipsoid    %  Drag tensor for Stokes flow around ellipsoid
+tweezer.stokesdrag       %  Drag tensor for Stokes flow around particle

+multipole.tmatsphere;           %  T-matrix for sphere
+multipole.tmatellipsoid;        %  T-matrix for ellipsoid
+multipole.incoming;             %  Multipole expansion of incoming fields
+tweezer.scatterer               %  Optical T-matrix scatterer
+tweezer.griddedScattererSphere  %  Gridded interpolator for spherical scatterer
+tweezer.griddedScattererPol     %  Gridded interpolator for scatterer with polar symmetry
+tweezer.walkersphere            %  Walker for spherical particles
+tweezer.walkerparticle          %  Walker for particle with polar or no symmetry

+tweezer.rotation         %  Rotation matrix using quaternions
+tweezer.boundingbox      %  Bounding box with periodic boundary conditions in xy-plane
```

In the following we discuss the properties and methods in more detail.

tweezer.fluidsphere

This class accounts for fluidic forces on spheres, and can be used to propagate them in absence or presence of Brownian motion.

```
classdef fluidsphere
  %  Stokes drag and diffusion for sphere in fluid.
  properties
    diameter        %  hydrodynamic diameter
    vel             %  fluid velocity in z-direction
    eta = 9.544e-4  %  viscosity (Pa s)
    temp = 293      %  fluid temperature
  end
end
```

The meaning of the various properties is self-explanatory, `vel` is the velocity of the fluid in OF2i setups, to be given in ms^{-1}. Objects can be initialized either without (optical tweezers) or with (OF2i) the fluid velocity.

```
fluid = tweezer.fluidsphere( diameter );
fluid = tweezer.fluidsphere( diameter, vel );
```

Alternatively, the velocity or the other parameters can be set at a later stage, for instance through `fluid.vel=vel`. Objects of type `tweezer.fluidsphere` allow for the solution of

Newton's equation (2) of the main text. The velocity for a given force `ftot` in pico-Newton can be computed from

```
vel = drift( fluid, ftot );
```

In presence of Brownian motion, we can update the sphere position in a time interval `dt` using

```
pos = browniant( fluid, pos, ftot, dt );
```

Note that `pos` can be either a 1×3 vector for a single particle or a `n`$\times3$ array for multiple particles. In OF2i it is sometimes advantageous to propagate the spheres along z rather than in time. This can be done with

```
[ pos, dt ] = brownianz( fluid, pos, ftot, dz );
```

where `dt` is the time increment for the propagation over `dz`.

tweezer.fluidellipsoid

With this function one can intialize the fluidic forces for ellipsoidal particles.

```
fluid = fluidellipsoid( diameter, ratio );
fluid = fluidellipsoid( diameter, ratio, vel );
```

Here `diameter` is the diameter of the ellipsoid in xy-directions and `ratio` the axis ratio. An prolate ellipsoid then has a ratio larger than one. The function initializes an object `tweezer.fluidpol` with the same functionality as described next.

tweezer.fluidpol

This class accounts for fluidic forces on particles with rotational symmetry around the z-axis. We assume that both the translational and rotational diffusion matrix is diagonal, and we ignore direct couplings between the translational and rotational degrees of freedom. The functionality is very similar to `tweezer.fluidsphere`, but one additionally has to account for the rotational degrees of freedom.

```
classdef fluidpol
  %  Stokes drag and diffusion for particle with polar symmetry in fluid.
  properties
```

```
    drag            %  diagonal components of drag tensor
    vel             %  fluid velocity in z-direction
    eta = 9.544e-4  %  viscosity (Pa s)
    temp = 293      %  fluid temperature
  end
end
```

It can be initialized with

```
fluid = tweezer.fluidpol( drag );
fluid = tweezer.fluidpol( drag, vel );
```

Here `drag` is a structure with the elements `drag.tt` for the translational and `drag.rr` for the rotational degrees of freedom. See for instance the last line in `tweezer.dragellipsoid`. Upon initialization, the following methods are available

```
[ vel, omega ] = drift( fluid, rot, ftot, ntot );
[ pos, rot ] = browniant( fluid, pos, rot, ftot, ntot, dt );
[ pos, rot, dt ] = brownianz( fluid, pos, rot, ftot, ntot, dz );
```

Here `omega` is the angular velocity [see Eq. (3) of main text] and `ntot` the optical torque. The positions and orientations of the particles are given by `pos` and the rotation matrices `rot`. See also the description of `tweezer.rotation` given below.

Stokes drag for particles of arbitrary shape

For particles with arbitrary shape we have implemented the class `tweezer.fluidparticle` that has the same properties and methods as the `tweezer.fluidpol` class, however, the drag tensor must account for the various couplings between translation and rotation. It can be computed for particles of arbitrary shape by discretizing the boundary in the form of a `particle` object with vertices and faces, as described in [1] and the help pages under `Base classes > Particle`.

```
drag = stokesdrag( p );
```

`drag` is a structure with the elements `drag.tt`, `drag.tr`, `drag.rt`, and `drag.rr` for the couplings between translational and rotational degrees of freedom. Our computational approach follows the work of H. Power and G. Miranda, SIAM J. Appl. Math. 47, 689 (1987).

multipole.tmatsphere, multipole.tmatellipsoid

The toolbox provides a number of classes and functions for computing and loading T-matrices for particles with arbitrary geometries, as described in the help pages under `Maxwell solvers > Multipoles`. For spherical particles, we additionally provide the wrapper function

```
tmat = multipole.tmatsphere( mat1, mat2, diameter, k0 );
tmat = multipole.tmatsphere( mat1, mat2, diameter, k0, 'lmax', lmax );
```

`mat1` and `mat2` are the materials inside and outside the sphere, `diameter` is the sphere radius, and `k0` is the wavenumber of light in vacuum. Additionally, one can pass the maximal number of spherical degrees. If this parameter is not provided, the function uses the Wiscombe cutoff for selecting `lmax`. A similar function also exists for ellipsoids where one has to additionally provide the axis ratio.

```
tmat = multipole.tmatellipsoid( mat1, mat2, diameter, ratio, k0, 'lmax', lmax );
```

multipole.incoming

This class allows computing the coefficients of the incoming fields $q_{\rm inc}$, see Eq. (1) of main text, using as input the incoming electromagnetic fields.

```
classdef incoming < multipole.base
  %  Multipole expansion of incoming fields.
  properties
    mat       %  material properties of embedding medium
    k0        %  wavenumber of light in vacuum
    fun       %  incoming fields [e,h]=fun(pos,k0)
    a         %  TM incoming coefficients
    b         %  TE incoming coefficients
  end
end
```

`mat` is the embedding medium, `k0` is the wavenumber of light in vacuum, and `fun` is a function or functor that returns the incoming electromagnetic fields for given positions and `k0`. The object can be initialized with

```
qinc = multipole.incoming( mat, k0, fun, 'lmax', lmax, 'diameter', diameter );
```

Here lmax is the maximal degree for the expansion in spherical multipoles, on default the Wiscombe cutoff parameter is used, and diameter is the diameter for the evaluation of the coefficients, see [5, Eq. (E.5)].

After initialization, the multipole coefficients can be computed from

```
qinc = eval( qinc );
qinc = eval( qinc, shift );
qinc = eval( qinc, shift, rot );
```

With these calls, the multipole coefficients a, b are computed. One can also provide a single or multiple shift parameters to compute $q_{\rm inc}$ for a particle located at the corresponding shift positions. If one provides one or several (size 3×3×n) additional rotation matrices rot, the coefficients are computed for the rotated particles (in the particle frame). For tweezer applications one can also compute the coefficients at one position, e.g. the trap center, and translate and rotate them to other particle positions and orientations.

```
qinc = eval( qinc );
q = translate( qinc, shift );
q = translate( rotate( qinc, rot ), shift, 'same', 1 );
```

The 'same' argument is needed for multiple shift and translation arrays.

tweezer.scatterer

Optical forces and other properties, such as optical cross sections or the power radiated into some given solid angle, can be computed with the scatterer objects.

```
classdef scatterer
  %  Optical T-matrix scatterer.
  properties
    tmat      %  T-matrix for scatterer
    qinc      %  multipole coefficients for incoming fields
  end
end
```

They are initialized with

```
scatterer = tweezer.scatterer( tmat, qinc );
```

Here qinc is either an object of type multipole.incoming, for a discussion see above, or a functor, such as a multipole translation for a spherical particle via

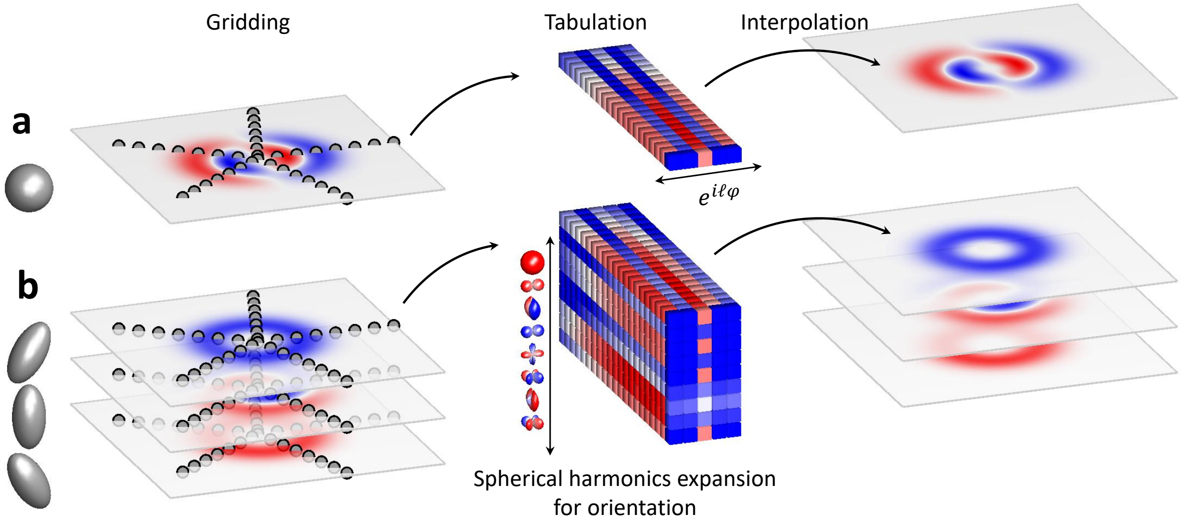

FIG. S1. (a) For spherical particles we provide `tweezer.griddedScattererSphere` objects, which compute optical forces and other user-defined functions on a $(r, \varphi, z = \text{const})$ or (r, φ, z) grid. We perform a Fourier transform along the azimuthal direction, and interpolate from the gridded data to the user-requsted positions. (b) A similar gridding can be done for particles with a polar symmetry, using objects of type `tweezer.griddedScattererPol`. Here we additionally perform an expansion in terms of spherical harmonics for the tabulation and interpolation of the particle orientations.

```
qinc = @( shift, ~ ) translate( qinc, shift );
```

After initialization, the optical forces and torques for given positions and rotations can be computed from

```
[ f, ~, sol ] = optforce( scatterer, pos );        %  spherical particle
[ f, n, sol ] = optforce( scatterer, pos, rot );   %  ellipsoidal particle
sol = solve( obj, pos );                           %  multipole solution
sol = solve( obj, pos, rot );
```

Note that for non-spherical particles the forces and torques are computed in the laboratory frame. `sol` it a multipole solution of type `multipole.solution`, which is centered at the particle position. A number of quantities can be computed from the solution vector, such as the scattered power.

```
sca = scattering( sol );
sca = scattering( sol, pinfty );
sca = scattering( sol, pinfty, 'shift', shift );
```

`pinfty` is a discretized boundary of type `particle`, which can be used to mimic the effect of a detector that covers only part of the solid angle (see also `trispheresegment`). The additional `shift` argument is needed to shift multipole solutions to the particle positions. One can also compute the fields and far-fields of a given solution through

```
[ e, h ] = fields( sol, pos );
[ e, h ] = farfields( sol, dir );
[ e, h ] = farfields( sol, dir, 'shift', shift );
```

`dir` are the directions of the optical far-fields.

tweezer.griddedScattererSphere

For simulations with many spheres (walkers) it might be beneficial to first compute the optical forces and other quantities on a grid, and to interpolate them at a later point.

```
classdef griddedScattererSphere
  %  Gridded force interpolator for spherical scatterer.
  properties
    scatterer %  optical scatterer
    r         %  radii for interpolation
    z         %  z-values for interpolation
    f         %  grid for optical force
    lmax      %  maximal order for azimuthal Fourier expansion
    fun       %  additional functions for gridding
  end
end
```

We use grids for the interpolation along the radial and propagation distances r and z, and perform a Fourier expansion along the azimuthal direction, see Fig. S1. For OF2i simulations it is usually beneficial to use a single z value, and to propagate all walkers along z. The class is initialized with

```
grid = tweezer.griddedScattererSphere( scatterer, r, z, 'lmax', lmax );
```

If `z` is a vector, a full three-dimensional table of optical forces and torques is computed, which can be time consuming. If `z` is a single value or empty `[]`, the computation is significantly faster. The evaluation of the optical force for given walker positions `pos` can be done with

```
f = optforce( grid, pos );
```

Note that for a single or empty `z` value all `pos(:,3)` must have the same value. It is also possible to tabulate and interpolate other quantities, such as the total scattered power. To this end, we must provide a function for the tabulation that is passed in the initialization

```
fun = @( sol, shift ) scattering( sol, 'shift', shift );
grid = tweezer.griddedScattererSphere( scatterer, r, 'lmax', lmax, 'fun', fun );
```

At a later stage, the scattered power can be obtained from

```
sca = feval( grid, pos );
```

One can also pass in the initialization multiple functions as a cell array. The evaluation `feval` then returns multiple output arguments.

tweezer.griddedScattererPol

Gridded tabulation and interpolation is also implemented for particles (walkers) with polar symmetry. Although all functions have been implemented in analogy to `tweezer.griddedScattererSphere`, we recommend using the object only for OF2i simulations with a single propagation distance z.

```
classdef griddedScattererPol
  %  Gridded force interpolator for scatterer with polar symmetry.
  properties
    scatterer %  optical scatterer
    r         %  radii for interpolation
    z         %  z-values for interpolation
    f         %  grid for optical force
    n         %  grid for optical torque
    lmax      %  maximal order for azimuthal Fourier and multipole expansion
    fun       %  additional functions for gridding
  end
end
```

The initialization and the computation of the optical force and torque is done with

```
grid = tweezer.griddedScattererPol( scatterer, r, z, 'lmax', lmax );
[ f, n ] = optforce( grid, pos, rot );
```

`lmax` is a vector with two entries, the first one controlling the azimuthal Fourier transform and the second one the spherical harmonics transformation of the rotational degrees of freedom. If both values are the same, one can also pass a single value. In general, we recommend using small values for `lmax`, say two or three. For a discussion of the other class methods interested readers are referred to the help pages.

tweezer.walkersphere

We provide wrapper classes for the evaluation of particle trajectories, which work fine in most cases of interest. In case of more specialized applications, for instance when users would like to compute non-standard quantities from the multipole solutions, we suggest using a more open loop structure, as discussed for the optical tweezer simulation at the beginning of this Supporting Information.

```
classdef walkersphere
  %  Walker for spherical particles.
  properties
    scatterer     %  optical force evaluator for spherical particle
    fluid         %  Stokes drag and diffusion for spherical particle
    pos           %  walker positions
    vel           %  walker velocity
  end
end
```

The class is initialized with

```
walker = tweezer.walkersphere( scatterer, fluid, pos );
```

`scatterer` is either a `tweezer.scatterer` or `tweezer.griddedScattererSphere` object. `pos` is the position of a single sphere (walker) or the positions of multiple spheres. For a time vector `tout` the walkers can be propagated without or with Brownian motion

```
wout = propagatet( walker, tout, 'waitbar', nt );
wout = browniant( walker, tout, 'nsub', nsub, 'waitbar', nt );
```

If the waitbar property is set, a waitbar shows the progress after `nt` time steps. `propagatet` uses an ODE solver of MATLAB, and `browniant` propagates the walkers using Gaussian random noise to mimic Brownian motion. It is possible to sub-divide each output time step into

`nsub` sub-intervals during evaluation. `wout` is a walker array that stores the walker positions and velocities at each time step.

For OF2i, we additionally provide a propagation along z rather in time. Let `zout` be an array where the walker positions and velocities are requested.

```
[ wout, tout ] = propagatez( walker, zout, 'waitbar', nz );
[ wout, tout ] = brownianz( walker, zout, 'nsub', nsub, 'waitbar', nz );
walker = trap( walker, 'dir', dir );
```

The last command provides the trapping positions of the walkers starting at `walker.pos`, which are allowed to move freely along `dir`.

tweezer.walkerparticle

A similar walker class has been implemented for walkers with polar symmetry or without any symmetry.

```
classdef walkerparticle
  %  Walker for particle in fluid.
  properties
    scatterer     %  optical force evaluator for particle
    fluid         %  Stokes drag and diffusion for particle
    pos           %  walker positions
    rot           %  rotation matrix for particle
    vel           %  walker velocity
  end
end
```

The class is initialized with

```
walker = tweezer.walkerparticle( scatterer, fluid, pos, rot );
walker = tweezer.walkerparticle( scatterer, fluid, pos );
walker.rot = rot;
```

The methods of the class are identical to those of `tweezer.walkersphere`, with the only difference that the `trap` function is not implemented.

Rotations

To set up rotation matrices, we provide the function

```
rot = rotation( t, 'order', order );
rot = rotation( t, 'order', order, 'angle', 'rad' );
```

Here `t` is an array of angles, given either in degrees (default) or radians (for `angle` property set), and `order` is the sequence of rotations such as `'zxz'`. The rotation angles and axes are processed form left to right. For instance, a random rotation matrix for `n` particles and size `3`×`3`×`n` is obtained through

```
rot = multipole.rotation( 360 * rand( n, 3 ), 'order', 'zxz' );
```

The toolbox internally also provides a class `tweezer.rotation` for the ODE solution of non-spherical particles.

tweezer.boundingbox

For the simulation of many walkers we provide a class that implements a bounding box with periodic boundary conditions in the transverse directions.

```
classdef boundingbox
  %  Bounding box with periodic boundary conditions in xy-plane.
  properties
    limits        %  minimal and maximal values for bounding box
  end
end
```

It is initialized with an array `limits=[xmin, xmax; ymin, ymax]` through

```
box = tweezer.boundingbox( limits );
```

The following methods have been implemented

```
a = area( box );             %  area of bounding box
pos = rand( box, n, z );     %  random initial positions at propagation distance z
pos = periodic( box, pos );  %  apply periodic boundary conditions
```

S2. VALIDATION OF TOOLBOX

In this section we demonstrate the accuracy of our NANOBEM toolbox for the computation of T-matrices and optical forces and torques.

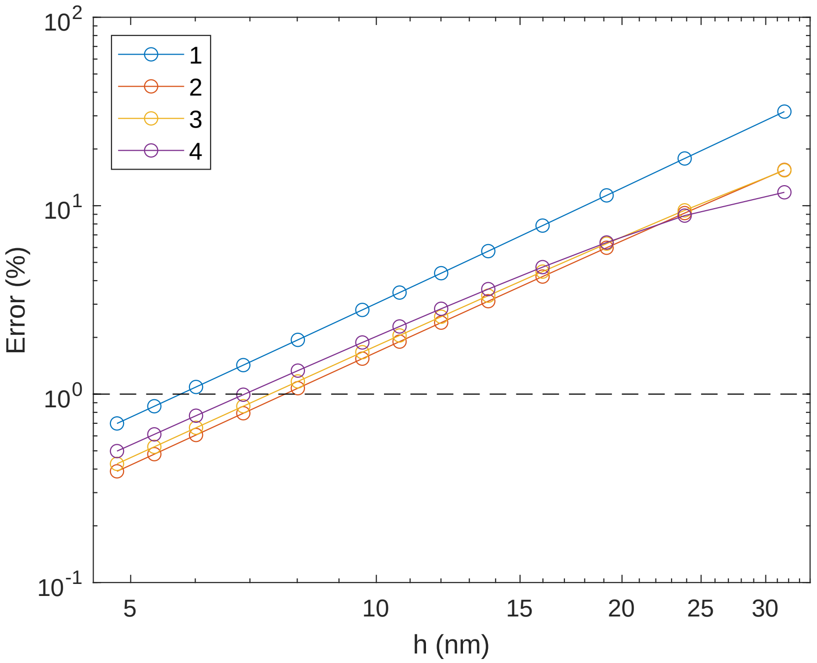

FIG. S2. Convergence of T-matrix elements for a nanosphere with a refractive index of $n = 3$, a diameter of 160 nm, for a vacuum wavelength of $\lambda = 500$ nm, and for different sphere discretizations with a mean edge length h. We plot for the magnetic multipole elements with $m = 0$ and for different spherical orders ℓ, indicated in the inset, the relative error with respect to the analytic Mie coefficients. With decreasing mesh size, the numerical results converge to the analytic Mie values.

T-matrices

The BEM implementation underlying NANOBEM is based on a Galerkin scheme that guarantees that the numerical solutions converge towards the true solutions for sufficiently fine boundary discretizations. This is demonstrated in the following for the computation of T-matrices. Ref. [6] suggests a data format for storing T-matrices, and brings together a variety of computational Maxwell solvers including NANOBEM for the computation of T-matrices. In [6] we also discuss how to compute T-matrices within the BEM approach.

Figure S2 investigates the accuracy of a T-matrix computed in NANOBEM for a nanopshere through comparison with the analytic Mie results. We use a sphere with a refractive index of $n = 3$ and a diameter of 160 nm, and for an excitation with a vacuum wavelength of $\lambda = 500$ nm. We show the relative error of the magnetic multipole elements for different sphere discretizations, characterized by the mean edge length h of the triangles of the discretized

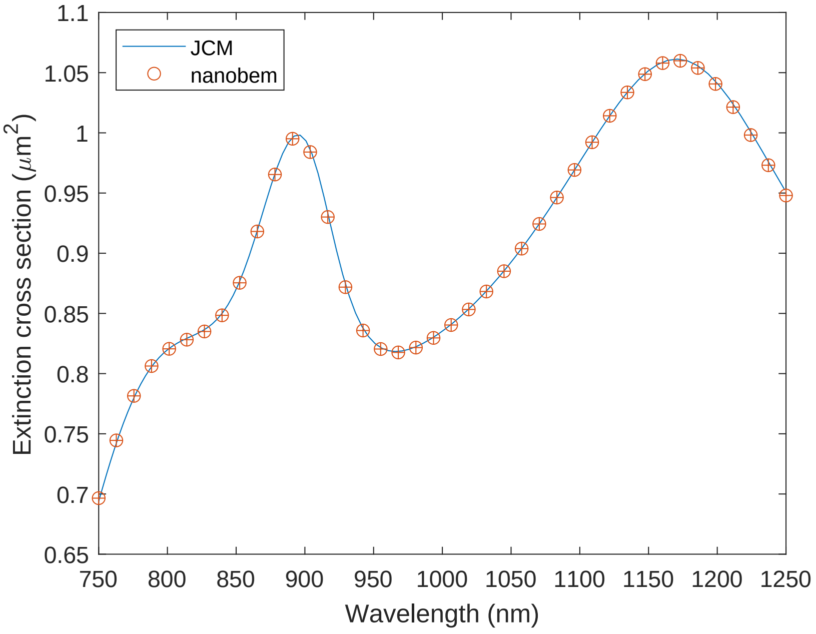

FIG. S3. Comparison between extinction spectra obtained with T-matrices which are computed with the JCM suite [6] and the NANOBEM toolbox for a TiO_2 cylinder.

sphere boundary. We observe that with decreasing h the various multipole coefficients converge towards the Mie results.

As a second nontrivial example we compare in Fig. S3 the optical extinction spectra for a TiO_2 cylinder with a diameter of 500 nm and a height of 300 nm, as obtained from the T-matrices computed with the JCM suite (solid line) and the NANOBEM toolbox (symbols), see Fig. 5 of [6] for details. In [6] this example has been selected for benchmarking T-matrix computations. In the figure we obtain perfect agreement with a boundary discretization of about 1500 triangles and for moderate runtimes of 15 minutes for the entire spectrum on a normal desktop computer, thus demonstrating the accuracy and efficiency for computing T-matrices with the NANOBEM toolbox. Additional demo programs can be also found in the help pages of the toolbox.

Optical forces and torques

To validate the computation of optical forces within the NANOBEM toolbox, we compare our resuls with the OTT toolbox [7] that is routinely employed in the optical tweezer community and can be downloaded from `https://ott.readthedocs.io/`. We consider a polystyrene

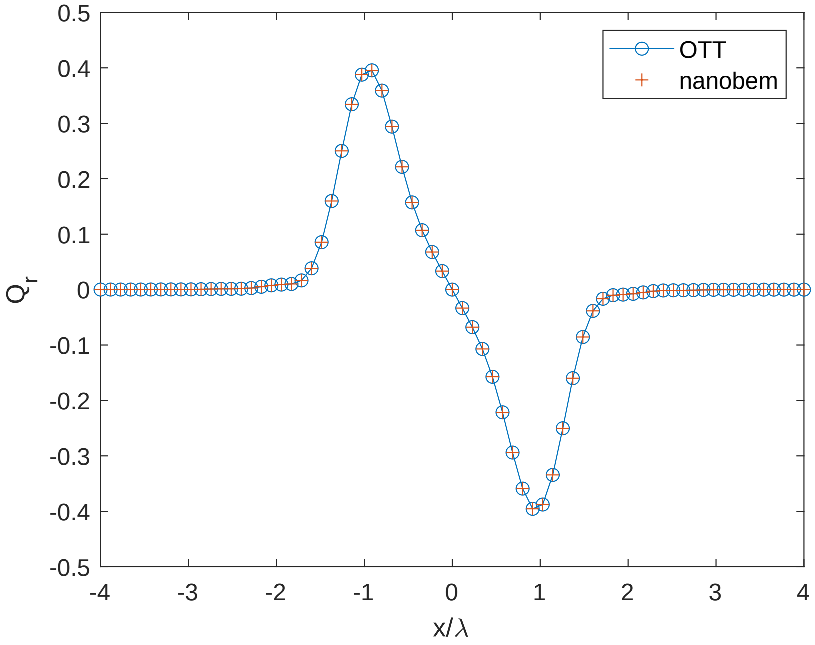

FIG. S4. Transverse trapping efficiency as computed with the OTT and NANOBEM toolboxes and for Gaussian beam.

sphere inside water, and compute the optical force using the following code (for details see OTT help pages).

```
n_medium = 1.33;       %  refractive index of embedding medium
n_particle = 1.59;     %  refractive index of sphere
lambda = 1064;         %  wavelength of light in vacuum
lambda1 = lambda / n_medium;

%  T-matrix for sphere
tmat = ott.Tmatrix.simple( 'sphere', lambda1, 'wavelength0', lambda, ...
    'index_medium', n_medium, 'index_particle', n_particle );
%  positions where field is evaluated
xout = linspace( -4, 4, 71 ) * lambda1;
posout = xout( : ) * [ 1, 0, 0 ] + [ 0, 0, 0 ];
%  Gauss beam
beam = ott.BscPmGauss( 'polarisation', [ 1, 1i ], 'NA', 1.02, ...
      'index_medium', n_medium, 'wavelength0', lambda, 'Nmax', 20, 'power', 1 );
%  optical force
fopt = transpose( ott.forcetorque( beam, tmat, 'position', posout .' ) );
```

Figure S4 compares the results for the transverse trapping efficiency `fopt(:,1)`, see also Fig. 1 of [7], obtained with the OTT and NANOBEM toolboxes for an incoming focused Gauss beam. In the NANOBEM simulations we use the same fields as for the OTT toolbox, which

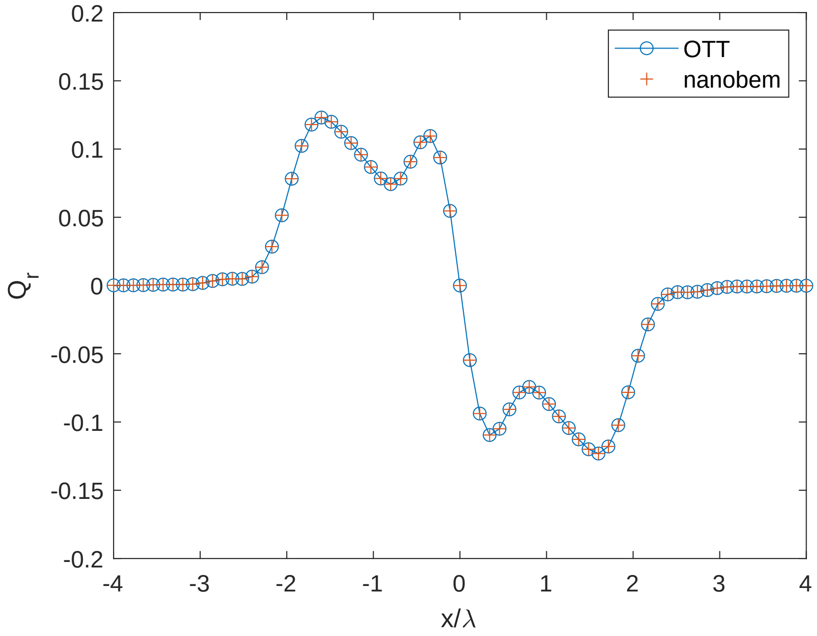

FIG. S5. Same as Fig. S4 but for Laguerre-Gauss beam.

we obtain apart from a constant prefactor from

```
[ e, h ] = beam.emFieldXyz( pos );
e = e / n_medium;
```

When using a Laguerre-Gauss beam through

```
%  Laguerre-Gauss beam
beam = ott.BscPmGauss( 'lg', [ 0, 2 ], 'polarisation', [ 1, 1i ], 'NA', 1.02, ...
      'index_medium', n_medium, 'wavelength0', lambda, 'Nmax', 20, 'power', 1 );
```

we obtain the results shown in Fig. S5. In both cases we observe perfect agreement between the different simulation softwares, thus demonstrating the proper implementation of optical forces in NANOBEM.

We finally investigate the optical forces and torques for a prolate polystyrene ellipsoid with a radius of 200 nm along the short axes, an axis ratio of 1 : 3, and for an incoming focused Gauss beam with the same parameters as stated above. Figures S6 and S7 show the force and torque efficiencies along the Cartesian axes, see [7] for a definition of the various quantities. Again we observe perfect agreement between the NANOBEM and OTT simulations.

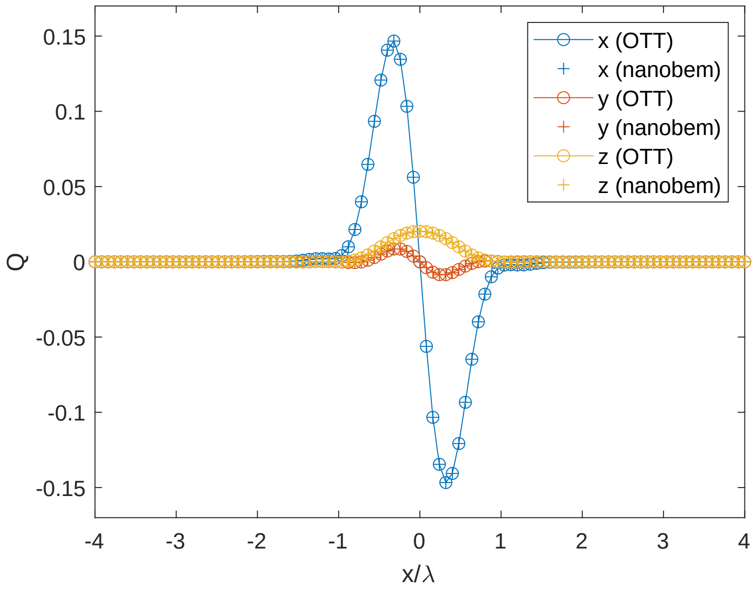

FIG. S6. Trapping efficiency for prolate polystyrene ellipsoid as computed with the OTT and NANOBEM toolboxes and for a focused Gauss beam.

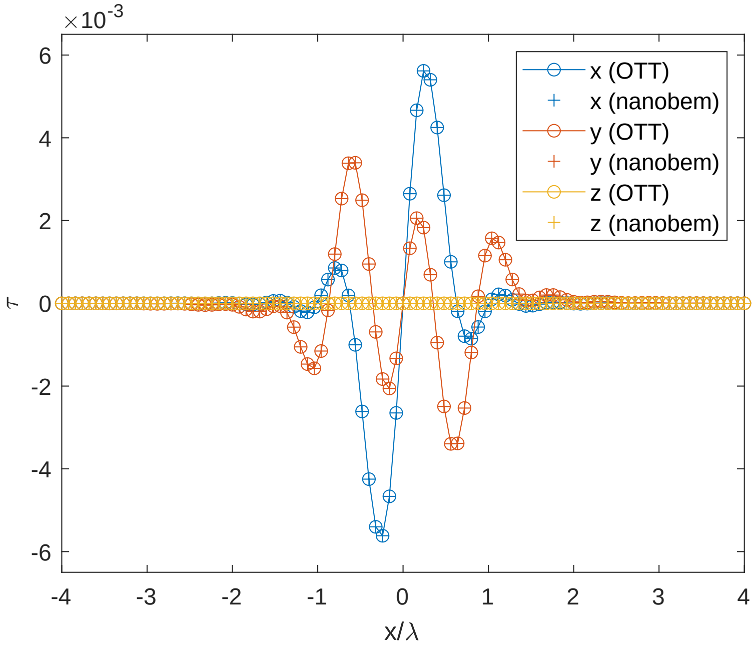

FIG. S7. Same as Fig. S6 but for torque efficiencies.

S3. THEORY

In this section we provide the working equations used in the NANOBEM toolbox for computing the optical forces and torques. We start by pondering on the multipole expansion of

the incoming and scattered electromagnetic fields. For the solenoidal solutions of the wave equation we introduce the vector spherical harmonics [8, Eq. (9.119]

$$\boldsymbol{X}_{\ell m}(\theta,\phi)=\frac{1}{\sqrt{\ell(\ell+1)}}\boldsymbol{L}Y_{\ell m}(\theta,\phi)\,, \tag{S1}$$

with the spherical harmonics $Y_{\ell m}$ for the angular degree ℓ and order m, respectively, and the angular momentum operator $\boldsymbol{L}=-i\boldsymbol{r}\times\nabla$. In the following we introduce for ℓ, m a single combined index n. The divergence-free, transverse solutions of Maxwell's equations can then be expanded in the basis [6]

$$\boldsymbol{M}_n^{(p)}=z_\ell^{(p)}(kr)\boldsymbol{X}_n(\hat{\boldsymbol{r}}) \tag{S2a}$$

$$\boldsymbol{N}_n^{(p)}=\frac{i}{k}\nabla\times\boldsymbol{M}_n^{(p)}(\boldsymbol{r})\,. \tag{S2b}$$

$z_\ell^{(p)}(kr)$ are spherical Bessel ($p{=}1$) or Hankel ($p{=}3$) functions that depend on the product of the wavenumber k and the distance from the origin r. One can easily check that the transverse vector function $\boldsymbol{N}_n^{(p)}$ fulfills the relation

$$-\frac{i}{k}\nabla\times\boldsymbol{N}_n^{(p)}=-\frac{1}{k^2}\nabla\times\nabla\times\boldsymbol{M}_n^{(p)}=\boldsymbol{M}_n^{(p)}\,. \tag{S3}$$

We next expand the incoming fields using spherical Bessel functions, which remain finite at the origin,

$$\boldsymbol{E}_i=\sum_n\left(a_n\boldsymbol{M}_n^{(1)}+b_n\boldsymbol{N}_n^{(1)}\right) \tag{S4a}$$

$$\boldsymbol{H}_i=Z^{-1}\sum_n\left(b_n\boldsymbol{M}_n^{(1)}-a_n\boldsymbol{N}_n^{(1)}\right)\,. \tag{S4b}$$

The incoming fields are then fully characterized by the coefficients a_n, b_n. For the scattered fields we use spherical Hankel functions, which become outgoing spherical waves at large distances,

$$\boldsymbol{E}_s=\sum_n\left(p_n\boldsymbol{M}_n^{(3)}+q_n\boldsymbol{N}_n^{(3)}\right) \tag{S5a}$$

$$\boldsymbol{H}_s=Z^{-1}\sum_n\left(q_n\boldsymbol{M}_n^{(3)}-p_n\boldsymbol{N}_n^{(3)}\right)\,. \tag{S5b}$$

The scattered fields are fully characterized by the coefficients p_n, q_n. We next derive the expressions for the asymptotic fields at large distances. The asymptotic expressions for the spherical Bessel and Hankel functions are [8, Eq. (9.89)]

$$j_\ell(x)\to\frac{1}{x}\sin\left(x-\frac{\ell\pi}{2}\right)=\frac{1}{2}\left((-i)^{\ell+1}\frac{e^{ix}}{x}-i^{\ell-1}\frac{e^{-ix}}{x}\right)$$
$$h_\ell^{(1)}(x)\to(-i)^{\ell+1}\frac{e^{ix}}{x}\,. \tag{S6}$$

We define the out- and in-going spherical waves

$$\phi_\ell^\pm(x) = (\mp i)^{\ell\pm 1}\frac{e^{\pm ix}}{x}\,. \tag{S7}$$

For large x values and neglecting contributions of the form $1/x^2$ we then obtain

$$\frac{d}{dx}\phi_\ell^\pm(x) \to \pm i\,\phi_\ell^\pm(x)$$
$$\frac{i}{k}\nabla\times\phi_\ell^\pm(x)\boldsymbol{X}_n \to \mp\phi_\ell^\pm(x)\hat{\boldsymbol{r}}\times\boldsymbol{X}_n\,.$$

The asymptotic incoming fields then become $\frac{1}{2}(\boldsymbol{E}_i^+ - \boldsymbol{E}_i^-)$ and $\frac{1}{2}(\boldsymbol{H}_i^+ - \boldsymbol{H}_i^-)$ with

$$\boldsymbol{E}_i^\pm = \sum_n\left(a_n\boldsymbol{X}_n \mp b_n\hat{\boldsymbol{r}}\times\boldsymbol{X}_n\right)\phi_\ell^\pm \tag{S8a}$$
$$\boldsymbol{H}_i^\pm = Z^{-1}\sum_n\left(b_n\boldsymbol{X}_n \pm a_n\hat{\boldsymbol{r}}\times\boldsymbol{X}_n\right)\phi_\ell^\pm\,. \tag{S8b}$$

Similarly, the asymptotic scattered fields are

$$\boldsymbol{E}_s^+ = \sum_n\left(p_n\boldsymbol{X}_n - q_n\hat{\boldsymbol{r}}\times\boldsymbol{X}_n\right)\phi_\ell^+ \tag{S9a}$$
$$\boldsymbol{H}_s^+ = Z^{-1}\sum_n\left(p_n\boldsymbol{X}_n + q_n\hat{\boldsymbol{r}}\times\boldsymbol{X}_n\right)\phi_\ell^+\,. \tag{S9b}$$

In what follows, we discuss how to compute the optical forces and torques for a nanoparticle excited by an incoming field characterized by the coefficients a_n, b_n. We assume that the coefficients q_n, p_n are at hand, either from the solution of the Mie or T-matrix equations. The transfer of optical momentum or angular momentum from the light fields to the nanoparticle can be computed from Maxwell's stress tensor [5, Eq. (4.35)]

$$\langle T_{ij}\rangle = \frac{1}{2}\mathrm{Re}\left[\varepsilon E_iE_j^* + \mu H_iH_j^* - \frac{1}{2}\delta_{ij}\left(\varepsilon|\boldsymbol{E}|^2 + \mu|\boldsymbol{H}|^2\right)\right]\,, \tag{S10}$$

which has been averaged over one oscillation period. The optical force $\boldsymbol{F}_{\rm opt}$ and torque $\boldsymbol{M}_{\rm opt}$ can be computed by integrating Maxwell's stress tensor over a closed boundary $\partial\Omega$,

$$\boldsymbol{F}_{\rm opt} = \oint_{\partial\Omega}\langle\overleftrightarrow{T}\rangle\cdot\hat{\boldsymbol{n}}\,dS \tag{S11a}$$
$$\boldsymbol{N}_{\rm opt} = \oint_{\partial\Omega}\boldsymbol{r}\times\langle\overleftrightarrow{T}\rangle\cdot\hat{\boldsymbol{n}}\,dS\,. \tag{S11b}$$

Here $\hat{\boldsymbol{n}}$ is the outer surface normal of the boundary and dS an infinitesimal boundary element. The above expressions account for the transfer of momentum or angular momentum from the light field to the nanoparticle.

Optical force from far-fields

We next evaluate Eq. (S11) in the optical far-field by integrating over a sphere at a sufficiently large distance r. Since in the far-field zone the electromagnetic fields are oriented perpendicularly to the propagation direction $\hat{\boldsymbol{r}}$, we have $\hat{\boldsymbol{r}}\cdot\boldsymbol{E} = \hat{\boldsymbol{r}}\cdot\boldsymbol{H} = 0$ and correspondingly only the diagonal term of Maxwell's stress tensor in Eq. (S10) contributes,

$$\boldsymbol{F}_{\text{opt}} = -\frac{1}{4}\oint_{\partial\Omega}\big(\varepsilon|\boldsymbol{E}|^2 + \mu|\boldsymbol{H}|^2\big)\,\hat{\boldsymbol{r}}\,dS\,. \tag{S12}$$

We next decompose the fields into incoming and scattered ones, and neglect the contribution $\varepsilon|\boldsymbol{E}_i|^2 + \mu|\boldsymbol{H}_i|^2$ because for the incoming fields momentum is a conserved quantity. Correspondingly, the term in parentheses of Eq. (S12) can be simplified to

$$\varepsilon|\boldsymbol{E}_s|^2 + \mu|\boldsymbol{H}_s|^2 + 2\text{Re}\big[\varepsilon\boldsymbol{E}_i\cdot\boldsymbol{E}_s^* + \mu\boldsymbol{H}_i\cdot\boldsymbol{H}_s^*\big]\,. \tag{S13}$$

Because the electromagnetic fields form together with the propagation direction a triad in the optical far-field, we have for the incoming fields $Z\boldsymbol{H}_i^\pm = \pm\hat{\boldsymbol{r}}\times\boldsymbol{E}_i^\pm$ and for the scattered fields $Z\boldsymbol{H}_s^+ = \hat{\boldsymbol{r}}\times\boldsymbol{E}_s^+$. With this, the mixed contribution in the brackets of Eq. (S13) can be simplified using

$$\frac{1}{2}\big(\boldsymbol{E}_i^+ - \boldsymbol{E}_i^-\big)\cdot\boldsymbol{E}_s^{+\,*} + \hat{\boldsymbol{r}}\times\frac{1}{2}\big(\boldsymbol{E}_i^+ + \boldsymbol{E}_i^-\big)\cdot\hat{\boldsymbol{r}}\times\boldsymbol{E}_s^{+\,*} = \boldsymbol{E}_i^+\cdot\boldsymbol{E}_s^{+\,*}\,.$$

Importantly, we observe that only the outgoing spherical waves contribute to the optical force while the contributions for the ingoing waves equate to zero. With the expression $\mu|\boldsymbol{H}_s^+|^2 = \varepsilon|\boldsymbol{E}_s^+|^2$ for the optical far-fields we are led to

$$\boldsymbol{F}_{\text{opt}} = -\frac{\varepsilon r^2}{2}\oint\text{Re}\left(|\boldsymbol{E}_s^+|^2 + \boldsymbol{E}_i^+\cdot\boldsymbol{E}_s^{+\,*}\right)\hat{\boldsymbol{r}}\,d\Omega\,, \tag{S14}$$

where the integral extends over the unit sphere. To arrive at our final expression, we insert the far-fields of Eqs. (S8,S9) into Eq. (S14),

$$k^2r^2\big(|\boldsymbol{E}_s^+|^2 + \boldsymbol{E}_i^+\cdot\boldsymbol{E}_s^{+\,*}\big) = \sum_{n,n'}\Big[\big(\bar{p}_n\boldsymbol{X}_n - \bar{q}_n\hat{\boldsymbol{r}}\times\boldsymbol{X}_n\big)\cdot\big(\bar{p}_{n'}^*\boldsymbol{X}_{n'}^* - \bar{q}_{n'}^*\hat{\boldsymbol{r}}\times\boldsymbol{X}_{n'}^*\big) + \big(\bar{a}_n\boldsymbol{X}_n - \bar{b}_n\hat{\boldsymbol{r}}\times\boldsymbol{X}_n\big)\cdot\big(\bar{p}_{n'}^*\boldsymbol{X}_{n'}^* - \bar{q}_{n'}^*\hat{\boldsymbol{r}}\times\boldsymbol{X}_{n'}^*\big)\Big]\,.$$

Here we have absorbed the phase factors from the out-going spherical waves into the multipole coefficients through $\bar{a}_n = (-i)^{\ell+1}a_n$, with corresponding definitions for the other incoming and scattered multipole coefficients. With $\hat{\boldsymbol{r}}\times\boldsymbol{X}_n\cdot\hat{\boldsymbol{r}}\times\boldsymbol{X}_{n'}^* = \boldsymbol{X}_n\cdot\boldsymbol{X}_{n'}^*$ and the relation

$\boldsymbol{X}_n \cdot \hat{\boldsymbol{r}} \times \boldsymbol{X}_{n'}^* = -\hat{\boldsymbol{r}} \times \boldsymbol{X}_n \cdot \boldsymbol{X}_{n'}^*$ we can then express the optical force in terms of the field coefficients through

$$\boldsymbol{F}_{\text{opt}} = -\frac{\varepsilon}{2k^2}\sum_{n,n'}\text{Re}\Big[\left(\oint \boldsymbol{X}_n \cdot \boldsymbol{X}_{n'}^* \,\hat{\boldsymbol{r}}\, d\Omega\right)\big(\bar{p}_n\bar{p}_{n'}^* + \bar{q}_n\bar{q}_{n'}^* + \bar{a}_n\bar{p}_{n'}^* + \bar{b}_n\bar{q}_{n'}^*\big) + \left(\oint \hat{\boldsymbol{r}} \times \boldsymbol{X}_n \cdot \boldsymbol{X}_{n'}^* \,\hat{\boldsymbol{r}}\, d\Omega\right)\big(\bar{p}_n\bar{q}_{n'}^* - \bar{q}_n\bar{p}_{n'}^* + \bar{a}_n\bar{q}_{n'}^* - \bar{b}_n\bar{p}_{n'}^*\big)\Big]\,. \tag{S15}$$

Optical torque from far-fields

With a similar approach we can compute the optical torque. Using in Eq. (S11b) for the integration boundary a sphere at large distances, we get

$$\boldsymbol{N}_{\text{opt}} = \frac{1}{2}\oint \text{Re}\big(\hat{\boldsymbol{r}} \times [\varepsilon \boldsymbol{E}(\boldsymbol{r}\cdot\boldsymbol{E}^*) + \mu \boldsymbol{H}(\boldsymbol{r}\cdot\boldsymbol{H}^*)]\big)\, dS\,. \tag{S16}$$

When separating the fields into incoming and scattered parts, we can exploit again that the contribution with solely incoming fields vanishes because of angular momentum conservation. From Maxwell's equations we find [8, Eq. (9.114)]

$$\begin{aligned} Zk\,\boldsymbol{r}\cdot\boldsymbol{H} &= -i\boldsymbol{r}\cdot\nabla\times\boldsymbol{E} = \quad \boldsymbol{L}\cdot\boldsymbol{E} \\ Z^{-1}k\,\boldsymbol{r}\cdot\boldsymbol{E} &= +i\boldsymbol{r}\cdot\nabla\times\boldsymbol{H} = -\boldsymbol{L}\cdot\boldsymbol{H}\,. \end{aligned} \tag{S17}$$

The term in parenteses of Eq. (S16) is then proportional to $\hat{\boldsymbol{r}} \times \boldsymbol{E}(\boldsymbol{L}\cdot\boldsymbol{H}^*) + \hat{\boldsymbol{r}} \times \boldsymbol{H}(\boldsymbol{L}\cdot\boldsymbol{E}^*)$. Together with the far-field relations for the electromagnetic fields we then get

$$\begin{aligned} \boldsymbol{N}_{\text{opt}} = \frac{\varepsilon}{2k}\oint \text{Re}\bigg(&\frac{Z^2}{2}\big[\boldsymbol{H}_i^{(+)} - \boldsymbol{H}_i^{(-)}\big]\boldsymbol{L}\cdot\boldsymbol{H}_s^{(+)\,*} - \frac{1}{2}\big[\boldsymbol{E}_i^{(+)} - \boldsymbol{E}_i^{(-)}\big]\boldsymbol{L}\cdot\boldsymbol{E}_s^{(+)\,*} - \\ &\frac{Z^2}{2}\boldsymbol{L}\cdot\big[\boldsymbol{H}_i^{(+)} - \boldsymbol{H}_i^{(-)}\big]\boldsymbol{H}_s^{(+)\,*} - \frac{1}{2}\boldsymbol{L}\cdot\big[\boldsymbol{E}_i^{(+)} - \boldsymbol{E}_i^{(-)}\big]\boldsymbol{E}_s^{(+)\,*} - \\ &\frac{Z^2}{2}\boldsymbol{H}_s^{(+)}\big[\boldsymbol{L}\cdot\boldsymbol{H}_s^{(+)\,*}\big] - \frac{1}{2}\boldsymbol{E}_s^{(+)}\big[\boldsymbol{L}\cdot\boldsymbol{E}_s^{(+)\,*}\big]\bigg)\, dS\,. \end{aligned}$$

Similarly to the discussion of the optical force, one can show that in the above expression the terms with $\boldsymbol{E}_i^{(-)}$, $\boldsymbol{H}_i^{(-)}$ equate to zero. We further note that $\boldsymbol{L}\cdot\boldsymbol{X}_n = \sqrt{\ell(\ell+1)}Y_n$ and $\boldsymbol{L}\cdot\hat{\boldsymbol{r}}\times\boldsymbol{X}_n = 0$. With this we are led to our final expression

$$\boldsymbol{N}_{\text{opt}} = -\frac{\varepsilon}{2k^3}\sum_{n,n'}\text{Re}\Big[\left(\oint Y_n\big(\boldsymbol{L}Y_{n'}^*\big)\, d\Omega\right)\big(\bar{p}_n\bar{p}_{n'}^* + \bar{q}_n\bar{q}_{n'}^* + \bar{a}_n p_{n'}^* \bar{+} b_n\bar{q}_{n'}^*\big) - \left(\oint Y_n\big(\hat{\boldsymbol{r}}\times\boldsymbol{L}Y_{n'}^*\big)\, d\Omega\right)\big(\bar{p}_n\bar{q}_{n'}^* - \bar{q}_n\bar{p}_{n'}^*\big)\Big]\,. \tag{S18}$$

Quite generally, all integrals in Eqs. (S15,S18) could be solved analytically using the well-known properties for products of spherical harmonics. However, in our computational approach we follow [9] and exploit that for appropriately chosen Gauss-Legendre integration points and weights the integrals can be evaluated numerically with numerical errors of the order of machine precision. In the NANOBEM toolbox the integrals are evaluated only once during the initialization of the `tweezer.scatterer` objects. This leads to a fast and accurate evaluation scheme for optical forces and torques.

REFERENCES

[1] U. Hohenester, N. Reichelt, and G. Unger, Nanophotonic resonance modes with the nanobem toolbox, Comp. Phys. Commun. **276**, 108337 (2022).

[2] U. Hohenester, Nanophotonic resonators in stratified media with the nanobem toolbox, Comp. Phys. Commun. **294**, 108949 (2024).

[3] F. Hitzelhammer, A. Dostalova, I. Zykov, B. Platzer, C. Conrad-Billroth, T. Juffmann, and U. Hohenester, Unified simulation platform for interference microscopy, ACS Photonics **11**, 2745 (2024).

[4] M. Šimić, C. Hill, and U. Hohenester, Theoretical description of optofluidic force induction, Physical Review Applied **19**, 034041 (2023).

[5] U. Hohenester, *Nano and Quantum Optics* (Springer, Cham, Switzerland, 2020).

[6] N. Asadova, K. Achouri, K. Arjas, B. Augui, R. Aydin, A. Baron, D. Beutel, B. Bodermann, K. Boussaoud, S. Burger, M. Choi, K. M. Czajkowski, A. B. Evlyukhin, A. Fazel-Najafabadi, I. Fernandez-Corbaton, P. Garg, D. Globosits, U. Hohenester, H. Kim, S. Kim, P. Lalanne, E. C. Le Ru, J. Meyer, J. Mun, L. Pattelli, L. Pflug, C. Rockstuhl, J. Rho, S. Rotter, B. Stout, P. Trm, J. O. Trigo, F. Tristram, N. L. Tsitsas, R. Valle, K. Vynck, T. Weiss, P. Wiecha, T. Wriedt, V. Yannopapas, M. A. Yurkin, and G. P. Zouros, T-matrix representation of optical scattering response: Suggestion for a data format, Journal of Quantitative Spectroscopy and Radiative Transfer **333**, 109310 (2025).

[7] T. A. Nieminen, V. L. Loke, A. B. Stilgoe, G. Knöner, A. M. Branczyk, N. R. Heckenberg, and H. Rubinsztein-Dunlop, Optical tweezers computational toolbox, J. Opt. A: Pure Appl. Opt. **9**, S196 (2007).

[8] J. D. Jackson, *Classical Electrodynamics* (Wiley, New York, 1999).

[9] I. N. Kozin, J. Tennyson, and M. M. Law, Effective computation of matrix elements between polynomial basis functions, Comp. Phys. Commun. **165**, 10 (2005).
